# Impact of eliminating retirement earnings test on labor supply and pension benefit claims

**DOI:** 10.1371/journal.pone.0304458

**Published:** 2024-08-09

**Authors:** Tomoki KITAMURA, Yoshimi ADACHI

**Affiliations:** 1 Department of Finance, Musashi University, Tokyo, Japan; 2 Faculty of Economics, Konan University, Hyogo, Japan; Universidade Federal de Minas Gerais, BRAZIL

## Abstract

This study explores the hypothetical elimination of Japan’s retirement earnings test (ET) for public pensions, focusing on its implications for older workers’ labor supply and pension-claiming behaviors. The ET currently reduces public pension benefits for individuals aged 65 and older if their earnings exceed specified thresholds, potentially discouraging employment in this demographic. Notably, the Japanese ET influences both immediate and future pension benefits, thus diminishing current payouts for working pensioners and foregoing beneficial actuarial adjustments—adjustments based on actuarial calculations that would otherwise increase future benefits to account for delayed pension claims. This dual impact may discourage the labor supply and influence pension-claiming behavior among older workers. Through a survey-based experiment with male workers aged 40–59 years expected to face the ET upon retirement, we assess three reform scenarios as the first study in the literature: (1) eliminating future benefit reductions through actuarial adjustments, thereby enhancing the value of deferred pension claims; (2) removing immediate benefit suspensions to increase current pension payments directly; and (3) a comprehensive reform combining both approaches. Our findings reveal that eliminating reductions through actuarial adjustments increases the intensive margin (labor hours and income) and encourages delayed pension claims. Conversely, removing immediate benefit suspensions influences both the extensive margin (decision to work) and the intensive margin but leads to earlier pension claims. By highlighting the importance of differentiating between immediate and future benefit components in designing ET reforms, this study demonstrates their significant impact on labor supply and pension-claiming decisions.

## Introduction

Global trends in population aging have raised concerns about the sustainability of post-retirement household incomes. Policymakers worldwide have formulated policies promoting household self-sustenance, including postponing retirement and delaying the public pension-claiming age. The retirement earnings test (ET), which reduces public pension benefits for those with higher labor income, discourages the labor supply of senior workers (e.g., Haider and Loughran [1]). We examine hypothetical situations to eliminate Japan’s current retirement ET rule (zaisyoku-rourei-nenkin in Japanese) and find new ways to promote continued employment and delayed public pension benefit claims for skilled senior male workers after the normal pension age (NPA) at which full public pension benefits are granted. Claiming for pension benefits does not necessarily imply retirement. A substantial proportion of pensioners worldwide, including Japan, work after retiring from their primary jobs and continue to access public pension benefits [2]. Reforming the ET may accelerate early public pension claims, which can reduce increased benefits based on actuarial adjustments and limit the standard of living at an advanced age [3]. We show that a particular type of ET elimination can promote labor supply and delay the public pension claiming age, which can promote policy discussions for future ET reforms.

In general, retirement ETs have tax and transfer features [4]. Tax features include threshold and withholding rates. If earnings exceed the threshold amount, public pension benefits are reduced by the withholding rate (*τ*). Meanwhile, the transfer feature increases reduced benefits as a future benefit stream based on actuarial adjustments. The Japanese ET has only tax features, whereas Social Security in the United States (U.S.) has both. Theoretically, an actuarially fair ET, such as that used in the U.S., would not affect labor supply if there is no liquidity constraint, and individuals are forward-looking and behave in a manner consistent with the life cycle model. However, recent empirical studies show that ET reforms have increased labor supply in the U.S. [4–6], the United Kingdom [7], and Canada [8]. In the case of only tax features, as in Japan, eliminating the ET is equivalent to reducing marginal tax rates. Changes in marginal tax rates depend on individual earnings levels. Indeed, the labor supply of those around the test threshold income increases due to the substitution effect, as explained later. Japanese empirical studies have shown mixed results regarding past ET reforms [2, 9, 10].

In Japan, while many senior workers continue to work after attaining NPA, a considerable labor shortage is expected owing to Japan’s declining birthrate and aging population [11–13]. Policies to secure labor are needed; if the ET discourages labor supply, its elimination deserves consideration. Although public pension benefits constitute the largest share of retirement income in Japanese households, their real benefits are expected to decline [14, 15]. These households are subject to longevity risk; if a household member exceeds the average life expectancy, their financial assets are likely to be depleted, leading to a reduced standard of living in their final years [16]. One solution to hedge longevity risk is to delay retirement and defer public pension benefit claims [17]. Deferring public pension claims increases benefits through actuarial adjustments, which can compensate for the decline in real benefits. If ET reform allows senior workers to work more and simultaneously delays the benefit-claiming age, realizing such a system would be of great social benefit to the economy.

Considering these challenges, this study bridges critical gaps in understanding retirement and labor policies in aging societies. We focus on two pivotal questions: How does eliminating the ET impact labor supply among Japan’s aging population, particularly those nearing retirement? Which ET reforms most effectively encourage sustained labor supply and the strategic delay of public pension benefits? These questions are central to policy reform debates, exploring the potential of removing financial disincentives to motivate continued workforce participation.

To this end, utilizing internet-based survey experiments, we investigate the effect of the hypothetical elimination of the current ET rules for those aged 65 years or older in Japan on male labor supply and public pension benefit-claiming behavior. Specifically, we analyze the hypothetical elimination of the ET regarding the responses of younger generations (aged 40–59 years) who need to plan their lives until retirement and are expected to face the ET. Examining their responses to the ET can be particularly helpful because eliminating the ET will probably not be implemented immediately, but over time. This generation is expected to become a policy target. Two types of ET rules are currently in operation: one for those aged 60–64 years, and the other for those aged 65 years or older. The former is to be discontinued; therefore, this study focuses on the latter. We examine whether they extend their employment and delay claiming public pension benefits, assuming they reach NPA (age 65). The Japanese ET only has tax features without transfer features and reduces current pension benefits if workers’ income exceeds a predetermined level. It also reduces actuarial adjustments, and thus future benefit streams, even if workers voluntarily delay the pension benefit-claiming age, as explained in detail in the next section. Hence, we examine three treatments representing different elimination patterns of the ET: (1) eliminating the reduction of actuarial adjustments, (2) eliminating the suspension of current benefits, and (3) simultaneously eliminating both elements. We examine the impact of these treatments on extensive (work or not) and intensive (change in income) margins.

We find that eliminating the ET promotes the labor supply after NPA, especially for those who choose low-income work (considered part-time). Treatments that eliminate the ET have heterogeneous effects. Eliminating the reduction in actuarial adjustments tends to increase high-income work (considered full-time) and delayed claiming of pension benefits, a novel finding that should be helpful in future policy discussions. By contrast, eliminating suspended current pension benefits is likely to increase both high-income work and early pension benefit claims. These results are consistent with the literature, showing that eliminating the ET encourages labor supply, and that pension-claiming behavior is determined based on the subjective evaluation of actuarial adjustments.

The main contribution of this study lies in its novel approach. First, we introduce new ways to eliminate the ET: ET elimination is divided into two elements in our treatment setting: eliminating the reduction in actuarial adjustments and the suspension of current benefits, which cannot be explicitly analyzed by empirical studies using observational data. Each factor may have a different effect on the extensive and intensive margins. Second, we analyze the responses of younger generations who are not currently subject to the ET, but are expected to face it. Our respondents were in their 40s and 50s. However, their behaviors cannot be analyzed using observational data. Third, we incorporate retirement and pension-claiming decisions and investigate whether they are associated with, or separate from, the ET.

Most evidence-based economic studies use actual decisions based on observational data. Researcher examining ET rule changes have utilized these observations to accurately measure the effect of rule changes. In contrast, this study leverages experimental surveys to explore respondents’ reactions to ET rule changes. We acknowledge the limitations, such as hypothetical bias [18–20], inherent in our method compared with observational data. Specifically, the actual behavior of respondents aged 65 years could diverge from their current responses owing to the absence of real-world consequences in our experimental setup.

Despite these limitations, our analysis is beneficial in measuring the effects of ET rule changes. Our method, which utilizes experimental surveys, has several advantages. First, we do not need to consider the endogeneity problems that exist in the empirical analyses of employment choices and pension benefit claims because of the experimental nature of our investigation. Second, it allows us to analyze the behavior of individuals in their 40s and 50s, which is unobservable in empirical data. Analyzing the behavior of these generations is critical for policy considerations regarding ET rule changes. Third, our recruitment strategy aimed to closely match the respondents’ attributes, such as family structure and annual income, with the assumptions of the experiment, thus enhancing the relevance of our findings. While recognizing the hypothetical nature of our experiment, we mitigated hypothetical bias by closely aligning the control setting with real-world situations and sequentially presenting treatments. Therefore, we believe that the treatment effects are measurable.

Several studies compare the hypothetical behavior reported in survey experiments with actual behavior and present evidence that the data generated through survey experiments are correlated with actual behavior [21, 22]. Brown et al. [23] show that the expected pension benefit claiming age correlates with the actual claiming age in the U.S. Health and Retirement Study. Survey experiments are widely used in retirement and pension-claiming decisions [24–27]. While we use experimental surveys to recognize the limitations associated with hypothetical scenarios, future research could benefit from conducting field experiments to further validate and build upon our findings. Such experiments offer a more direct method for observing actual behavior in real-world settings, thereby potentially providing more concrete evidence of the effectiveness of policy changes.

The results of this study have several international implications. Although Japan has the oldest population in the world and a well-established public pension system, maintaining a good standard of living based solely on the public pension system is challenging. Many developed countries, including the U.S., use the ET, which may restrict the supply of senior workers. A similar situation was observed in developing countries. To maintain a sustainable social security system, understanding how younger generations approaching retirement view the ET, and how they plan to work and receive pensions during retirement can be useful for researchers and policymakers worldwide.

The remainder of this paper is structured as follows. The subsequent section reviews the related literature. The background section provides an overview of Japan’s public pension system and the ET rule. The methods section elaborates on the survey design, theoretical predictions regarding ET elimination, and the empirical methods employed. The results section presents the primary findings and robustness checks. This is followed by a discussion on the policy implications of the results. The paper concludes with a final section summarizing the conclusions.

## Literature review

Recent empirical studies focusing on the U.S. have shown that relaxing ET rules increases labor supply. Friedberg [5] finds a significant effect of ET changes in 1978, 1983, and 1990 on the labor supply of older workers, especially for those just below the ET-exempt threshold. Loughran and Haider [6] report that ET elimination in 1983 and 2000 increased the male labor supply, especially for those aged 65–69 years. Song and Manchester [4] find that the elimination of the ET in 2000 increased earnings just around and above the threshold at which Social Security benefits were received. Haider and Loughran [1] conclude that, until 2000, the U.S. ET in operation significantly reduced the earnings of individuals over the full retirement age.

In the UK, Disney and Smith [7] report that eliminating the ET in 1989 increased work hours and earnings for both men and women. In Canada, Baker and Benjamin [8] find a shift from part-time to full-time work for men after the ET was eliminated because workers could not adjust the labor supply smoothly (labor market rigidities). Hernæs and Jia [28] examine the Norwegian threshold change for the 2002 ET reform. The Norwegian ET can be viewed as an implicit tax on earnings because it has no transfer feature similar to the Japanese ET. The authors find that the reform substantially affects retirees’ earnings distribution, which is strongest around the threshold level. Overall, these recent studies show that ET elimination significantly encourages labor supply. However, because each country has different public pension systems, ET rules, and working environments, these results cannot be directly applied to eliminate the ET in Japan.

Japanese studies have examined the effects of the ET and its reforms. Earlier studies focused on the ET in individuals in their early 60s [29–34]. Recently, more senior workers have continued to work beyond the age of 65. However, studies focusing on the ET in the late 60s are limited and the results are mixed [2, 10, 35]. Shimizutani and Oshio [9] examine the effect of eliminating the ET for those aged 65 years or older in 1985 and reinstating it in 2002. They find that ET elimination in 1985 encouraged the labor supply of senior male workers. However, the 2002 reinstation provides little evidence of affecting the male and female labor supply. The authors argue that their results differ because of labor market conditions. Specifically, in the mid-1980s, the Japanese economy was booming, and labor demand was strong. By contrast, the Japanese economy experienced deflation in the first half of the 2000s, and labor demand was weak. Oshio et al. [2] examine the effect of public pension-related policies on the labor supply of senior individuals. They consider the ET, raising the NPA, and wage subsidies for older workers. They find that raising NPA is the most effective solution for increasing male labor supply, followed by ET elimination. However, they use simulations based on regression analysis, which does not result from individual behaviors.

Since these studies, the labor market conditions in Japan have changed significantly. As explained in the following section, the NPA for public pension has increased from 60 to 65. Many Japanese employees continue to work part-time rather than quitting the labor force entirely after retiring from their primary full-time work [13, 35, 36]. This situation became prominent after the implementation of the senior worker employment policy (Act on Stabilization of Employment of Elderly Persons) in 2006. These changes require an examination of ET elimination in the current situation.

Studies examining the effect of the ET on public pension-claiming behaviors are limited. Gruber and Orszag [3] report that ET reform accelerates earlier Social Security benefit claims. It reduces benefit levels by giving up actuarial adjustments, and thus increases concerns about the standard of living in advanced age. Disney and Smith [7] argue that deferred pension benefits depend on individual evaluations of the actuarial fairness of delayed benefits. Those who expect longevity defer their claim age, even after the ET reform. However, there is room for research on the relationship between the elimination of the ET and pension-claiming behavior. This study examines a new method for ET elimination in Japan that can increase the labor supply and delay pension benefit claims.

## Background

### Public pension and senior worker employment rule in Japan

Japan’s public pension system consists of a basic pension (BP; first-tier, *kiso-nenkin* in Japanese) and employee pension insurance (EPI; second-tier, *kosei-nenkin* in Japanese). Self-employed persons and students over the age of 20 participate in the BP (also called the national pension or *kokumin-nenkin* in Japanese). The BP premium is fixed, and the benefits are adjusted proportionally based on the contribution periods. The BP premium is JPY 16,540 and its full benefit is JPY 65,141 per month in 2020, which requires 40 years of contribution. The average BP benefit is JPY 56,000 [37]. Participants with BP were excluded from the study. Private company employees and public servants participate in the EPI, which is our study target. After 2017, the premium is 18.3% of the remuneration, half of which is paid by employers. The EPI benefits are adjusted based on remuneration and length of contributions. The average EPI benefit is JPY 146,000, including the BP (fixed part of a pension) benefits [37]. The earliest age at which BP and EPI benefits can be claimed is 60 years, and the latest is 70 years (75 years after 2022).

The NPA is the standard starting age for public pension benefits defined by the government where full pension benefits can be received. The NPA of the BP and EPI has gradually increased. The NPA of BP for men increased from 60 to 61 in 2001 and continued increasing by one year every three years until it reached 65 in 2013. The NPA of EPI (wage-proportional part of the pension) increased from 60 to 61 in 2013 and will continue to rise by one year every three years until it reaches 65 in 2025. For women, the increase has been similar but with a five-year lag, which means that the NPA for the BP reached 65 in 2018 and will reach 65 by 2030 for the EPI. In addition to public pension, some private companies offer company pension plans (third-tier). A defined contribution pension plan was established in 2001, and a revised defined benefit pension plan was introduced in 2002. Individuals can also participate in the individual defined contribution (iDeCo) plan introduced in 2001.

The mandatory retirement age is the age at which a person must retire from the company, as defined by its employment rules; this age has been at least 60 years since 1998. The increase in NPA to 65 years may raise social concerns because household income may be unstable between the mandatory retirement age and NPA. The senior worker employment policy (Act on the Stabilization of Employment of Elderly Persons) was revised in 2006, and obligates employers to take at least one of the following three measures with certain exemptions: (1) raising the mandatory retirement age to the NPA, (2) setting up formal rules for employment extension and reemployment after the mandatory retirement age, or (3) abolishing the mandatory retirement age. The 2013 revision made this requirement mandatory. Generally, the mandatory retirement age and NPA are equal, or the NPA is higher. Our respondents were EPI beneficiaries aged 40–59 years. For most respondents, the NPA is 65, allowing them to continue working up to 65 if they choose, despite the mandatory retirement age being 60 years.

### Earnings test rule in Japan

The ET in Japan (*zaisyoku-rourei-nennkin*) reduces EPI benefits if total income (including wage income and EPI benefits) exceeds a certain threshold. The current ET for individuals aged 65 years or older was introduced in 2002. The threshold in 2022 was JPY 470,000 for total monthly income. We describe the current ET rule in Japan along with our experimental setting (for details, see Japan Pension Service [38]). Actual calculations are applied every month; however, in our experiment, the calculations are made on an annual basis. The annual suspended EPI benefit (*S*) at 65 years of age (NPA) is as follows:

$$S_{65}=\text{min}\{0.5*\text{max}\left(\frac{W_{65}+I_{65}}{12}-470000,\;0\right),\frac{I_{65}}{12}\}\times 12,$$ (1)

where *W* is the annual (adjusted standardized wage) income used to calculate the pension premium and *I* is the annual EPI benefit, where the subscripts indicate age. Eq (1) implies that half of the benefits above the threshold are reduced until all EPI benefits are suspended. BP benefits (*B*) are not subject to suspension. The annual total pension benefits (*P*), if received from the age of 65, is as follows:

$$P_{65}=B_{65}+I_{65}-S_{65}.$$ (2)

Even if pension benefits are voluntarily deferred, part of the actuarial adjustments is reduced for those earning more than the threshold. The pension benefit received from age 66 is as follows:

$$P_{66}=\left(1+g\right)P_{65}+S_{65},$$ (3)

where *g* is the actuarial adjustment rate (8.4% in 2022 after the NPA) and *P*_65_ is the pension benefit assumed to be received if claimed at age 65, as calculated by Eq (2). Eq (3) shows that no actuarial adjustment is applied to suspended benefits (*S*_65_). As of 2018, approximately 410,000 individuals (17% of current working beneficiaries) have suspended their benefits. Approximately 200,000 individuals (8% of current working beneficiaries) have suspended their full benefits [39].

The details of our experiment are explained in the following section, where we consider different ET-elimination patterns. First, we propose eliminating the reduction of future benefits by actuarial adjustment by setting S_*65*_ = 0 in Eqs (2) and (3); specifically, pension benefits at age 66 (delayed benefits) are calculated as follows:

$$P_{65}^{*}=B_{65}+I_{65}-0,$$ (4)

$$P_{66}=\left(1+g\right)P_{65}^{*}+0,$$ (5)

where $P_{65}^{*}$ is the unreceived (postponed) pension benefit at age 65. Second, we propose eliminating the suspension of immediate benefits by setting S_*65*_ = 0 in Eq (2):

$$P_{65}=B_{65}+I_{65}-0.$$ (6)

Although Eqs (4) and (6) may appear identical, they differ in the timing of the benefit claims. In Eq (4), benefits are not claimed at age 65 but are postponed to 66. Conversely, in Eq (6), benefits are claimed and received starting at the age of 65.

The other current ET rule in Japan states that, for those aged 60–64 years, the threshold is the same as that for those aged 65 years or older after 2022. This rule will be discontinued in 2025 for men and in 2030 for women when the NPA reaches 65. The ET rule for those aged 65 years or older applies to these workers even when they claim benefits before the age of 65. Therefore, we focus on the ET rule for those 65 or older.

## Methods

### Base experimental setting and control group

In our survey experiment, respondents faced a hypothetical situation regarding retirement and pension-claiming decisions, similar to discrete choice experiments [25, 40, 41]. Respondents were asked to assume they were 59 years old and to consider retiring and claiming public pension benefits at age 65 or 66. We constructed five situations (or work plans): Plans A–E. These work plans comprised labor income and pension benefits: labor income at ages 65 and 66 and pension benefits at ages 65 and 66 or older. Labor income included two types: high and low income. Pension benefits were based on current standard amounts, applying the ET rule. Specifically,

Plan A denotes high income with pension benefits (HIWP),
Plan B denotes high income without pension benefits (HINP),
Plan C denotes low income with pension benefits (LIWP),
Plan D denotes low income without pension benefits (LINP), and
Plan E denotes no work with pension benefits (No work), respectively.

We asked the respondents which work plan they preferred most. Oshio et al. [2] use similar categories to represent the work and public pension benefit receiving statuses of senior workers in Japan. We suppose that high income corresponds to full-time work and low income corresponds to part-time work (reduced hours). Baker and Benjamin [8] and Disney and Smith [7] show a discrete shift from part-time to full-time employment. In a Japanese study, Higuchi and Yamamoto [32] analyze the labor supply as the difference between full-time and part-time workers. This study interprets part-time as reemployment after the mandatory retirement age, resulting in reduced working hours.

In reality, decisions to receive pension benefits and work (retirement) are highly associated, but they can also be considered separate decisions under certain conditions. For example, even after the mandatory retirement age, individuals can be rehired to work or move to another company; and they can decide whether to receive their benefits. However, they generally receive benefits once they retire. For our experiment, we assume that the participants can separately decide on working and receiving benefits (Plans A–D), excluding the full retirement decision at age 65 (Plan E).

We designed one control and three experimental treatments to assess the impact of eliminating the ET. Tables 1–4 outlines the experimental instructions provided to respondents. Table 1 introduces the experimental parameters for the control group, representing the status quo and reflecting the conditions under the existing ET rule, as previously discussed. This setup forms the foundation of our experiment. Below, we detail the characteristics of each plan, focusing on income levels, pension-claiming ages, and pension benefits:

- **Income at age 65:** Plans A (HIWP) and B (HINP) are characterized by high incomes (annual JPY 6.65 million (M)), while Plans C (LIWP) and D (LINP) feature low incomes (JPY 4.43 M).
- **Pension claiming age (65 or 66)**: Plans A and C involve working until age 66 and receiving public pension benefits from age 65 (early pension claiming). In contrast, Plans B and D entail working until age 66 without receiving benefits at age 65 (delayed pension claiming). Plan E involves retiring at age 65 and receiving benefits from age 65.
- **Pension benefits at age 65:** Based on the Japan Pension Service [38], we set the EPI full annual benefits before the ET at age 65 (*I*_65_) as JPY 1.2 M and BP full annual benefits (*B*_65_) as JPY 0.78 M. The total full benefits before applying the ET rule sum to JPY 1.98 M. The benefits are suspended based on the ET rule. Under the ET rule, benefits in Plan A are reduced to JPY 0.88 M (= 0.78 M +1.2 M– 1.1 M) from Eq (2) due to high labor income. The suspended EPI benefits (*S*_65_) amount to JPY 1.1 M from Eq (1), given *W*_65_ = 6.65 M and *I*_65_ = 1.2 M. Plans C and E at age 65 receive full benefits (JPY 1.98 M) due to low labor income (*W*_65_ = 4.43 M).
- **Pension benefits at age 66:** Plan A has full benefits (JPY 1.98 M) because of no labor income or corresponding benefit suspension. Benefits for Plan C remain unchanged from age 65. For Plan B, benefits starting at age 66 (JPY 2.05 M) are lower than those for Plan D (JPY 2.15 M) due to decreased actuarial adjustments. From Eqs (2) and (3), the pension benefits at age 66 for Plan B are JPY 2.05 M = (1 + 8.4%) (0.78 M + 1.2 M– 1.1 M) + 1.1 M. Those of Plan D are JPY 2.15 M = (1 + 8.4%) (0.78 M +1.2 M).

**Table 1:** Experimental instruction and parameter settings for control group. Suppose you are now 59 years old. You are considering whether to retire at age 65 or work at age 65, and retire at age 66. Which work style and way of receiving the public pension (the sum of EPI and BP) would you prefer? Please choose the option that best applies to you.

		Plan A (HIWP)	Plan B (HINP)	Plan C (LIWP)	Plan D (LINP)	Plan E (No work)
Age 65	Labor income	665	665	443	443	0
	Pension benefits	88	0	198	0	198
Age 66 or older	Labor income	0	0	0	0	0
	Pension benefits	198	205	198	215	198
Your choice		〇	〇	〇	〇	〇
Unit: Annual JPY 10,000						
**Explanation of work plan**						
• In Plan A (HIWP: high-income with pension benefits), you obtain an annual labor income of JPY 6.65 M at 65 with annual public pension benefits of JPY 0.88 M. You retire at 66 and obtain public pension benefits of JPY 1.98 M annually.						
• In Plan B (HINP: high-income without pension benefits), you obtain an annual labor income of JPY 6.65 M at 65 without public pension benefits. You retire at 66 and obtain public pension benefits of JPY 2.05 M annually.						
• In Plan C (LIWP: low-income with pension benefits), you obtain an annual labor income of JPY 4.43 M at 65 by reducing work hours with annual public pension benefits of JPY 1.98 M. You retire at 66 and obtain annual public pension benefits of JPY 1.98 M.						
• In Plan D (LINP: low-income without pension benefits), you obtain an annual labor income of JPY 4.43 M at 65 by reducing work hours without public pension benefits. You retire at 66 and obtain annual public pension benefits of JPY 2.15 M.						
• In Plan E (No work), you retire at 65 and obtain annual public pension benefits of JPY 1.98 M.						

**Note**: JPY 10,000 is approximately USD 83.3. Thus, JPY 6,650,000, as shown in Plan A for labor income at age 65, is approximately USD 55,417. Symbols in parentheses, such as HIWP, are intended for readers of this paper. Such explanations were excluded from the survey. The actual explanation is provided in Japanese (the same applies to Tables 2–4).

**Table 2:** Enhancement of future pension (FP) treatment.

		Plan A	Plan B	Plan C	Plan D	Plan E
Age 65	Labor income	665	665	443	443	0
	Pension benefits	88	0	198	0	198
Age 66 or older	Labor income	0	0	0	0	0
	Pension benefits	198	**215**	198	215	198
Your choice		〇	〇	〇	〇	〇
Unit: Annual JPY 10,000						

**Note**: Explanations of work plans are provided, similar to Table 1. Full instructions are in Table A2 of Online Appendices in S1 Appendix.

**Table 3:** Enhancement of current pension (CP) treatment.

		Plan A	Plan B	Plan C	Plan D	Plan E
Age 65	Labor income	665	665	443	443	0
	Pension benefits	**198**	0	198	0	198
Age 66 or older	Labor income	0	0	0	0	0
	Pension benefits	198	205	198	215	198
Your choice		〇	〇	〇	〇	〇
Unit: Annual JPY 10,000						

**Note**: Explanations of work plans are provided, similar to Table 1. Full instructions are in Table A3 of Online Appendices in S1 Appendix.

**Table 4:** FPCP treatment.

		Plan A	Plan B	Plan C	Plan D	Plan E
Age 65	Labor income	665	665	443	443	0
	Pension benefits	**198**	0	198	0	198
Age 66 or older	Labor income	0	0	0	0	0
	Pension benefits	198	**215**	198	215	198
Your choice		〇	〇	〇	〇	〇
Unit: Annual JPY 10,000						

**Note**: Explanations of work plans are provided, similar to Table 1. Full instructions are in Table A4 of Online Appendices in S1 Appendix.

In addition to outlining these parameters, a brief description of each work plan is provided.

### Treatment groups

To examine the effect of ET elimination, we constructed three treatments in addition to the above control setting: enhancement of future pension (FP), enhancement of current pension (CP), and FPCP treatments. The FP treatment eliminated the reduction in actuarial adjustments, whereas the CP treatment eliminated the suspension of current (age 65) benefits. The FPCP treatment eliminated the reduction in both actuarial adjustments and current benefits, meaning that the ET was eliminated completely. Table 2 presents the FP treatment parameters. For the FP treatment, the reduction in future pension growth was eliminated. Actuarial adjustment (currently 8.4% annually) increases benefits if the benefit-claiming age is delayed after NPA. However, under the current ET rule, no actuarial adjustment was performed for a part of the benefits, as shown in Eq (3). The FP treatment eliminated this decreased actuarial adjustment, meaning that an individual could receive full delayed benefits at 66 years or older, regardless of labor income at 65 years, as shown in Eqs (4) and (5). The only difference between Tables 1 (control) and 2 (treatment FP) is the pension benefits for those aged 66 or older in Plan B. In Table 1, the benefits were JPY 2.05 M, whereas in Table 2, they were JPY 2.15 M because the reduction of the actuarial adjustment was eliminated.

Table 3 presents the CP treatment parameters. In the CP treatment, the suspension of current benefits was eliminated, meaning that full benefits at age 65 are obtained regardless of current labor income, as shown in Eq (6). The only difference between Tables 1 (control) and 3 (CP treatment) lies in the pension benefit for those over 65 years in Plan A. In Table 1, the benefits are JPY 0.88 M, whereas in Table 3, they are JPY 1.98 M due to the elimination of the benefit suspension at 65 years.

Table 4 shows the FPCP treatment parameters. In the FPCP, the suspension of both future and current pension benefits was simultaneously eliminated. That is, the ET was completely eliminated, and there was no reduction in current pension benefits or actuarial adjustments. Tables 1 (control) and 4 (treatment FPCP) differ in terms of pension benefits for individuals aged 65 years in Plan A and those aged 66 years or older in Plan B. In Table 4, the benefits at age 65 in Plan A are JPY 1.98 M, and the delayed benefits claimed at age 66 in Plan B are JPY 2.15 M.

Generally, controlling for the respondent’s situation in an experiment was preferable. For example, explaining the effect of early or late pension claims might have been necessary to make appropriate retirement decisions. However, additional explanations could have burdened the respondents. Thus, we provided only the minimum necessary explanations to our respondents; other situations were left to the respondents’ discretion.

### Theoretical and experimental predictions on ET elimination

**Fig 1:**
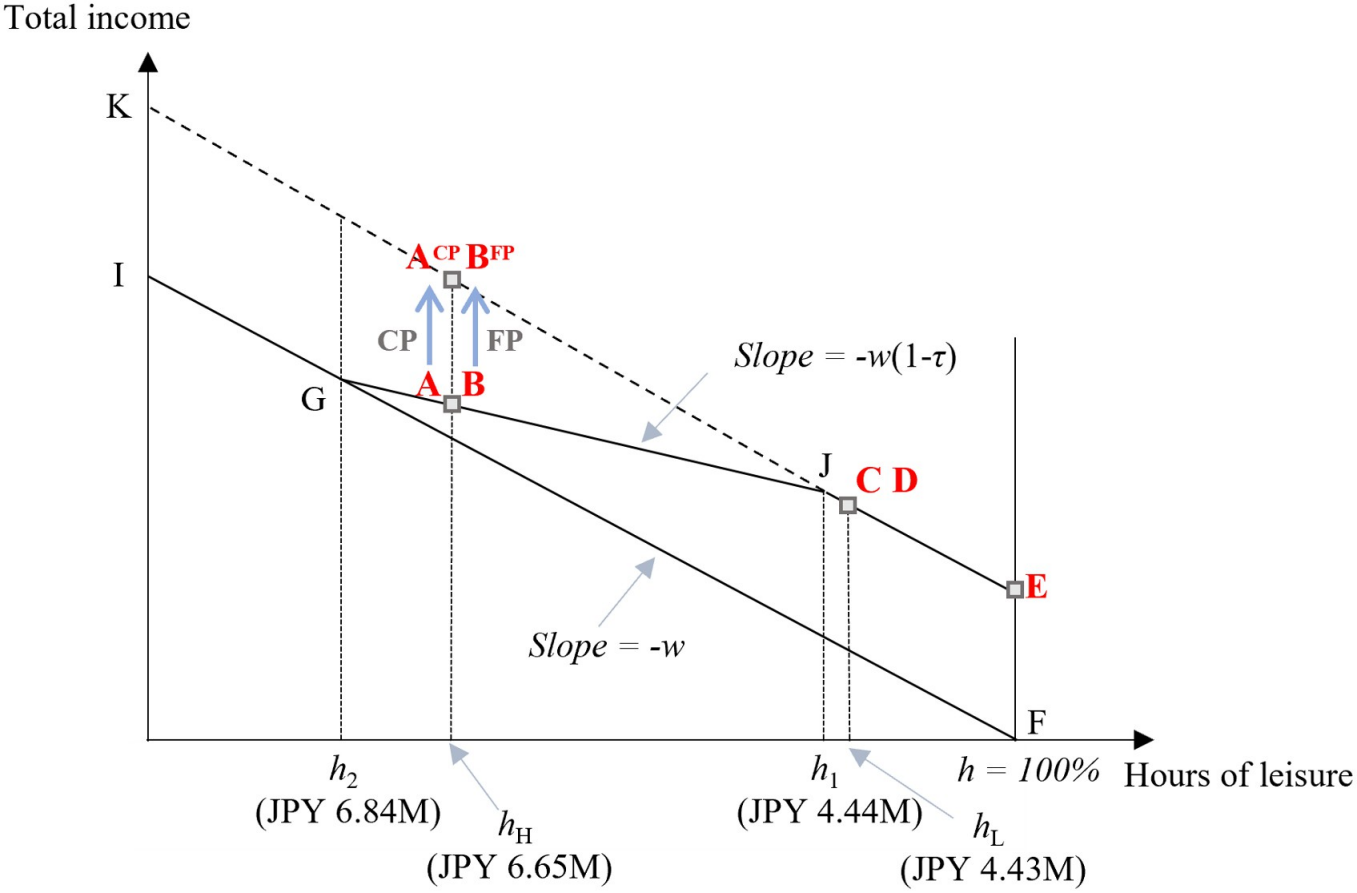
Retirement earnings test and budget constraint. **Note**: Plans C and D are in the same location because the only difference is the timing of the actuarially fair benefit claims. The same applies to Plans A and B and Plans A^CP^ and B^FP^. Here, *h*_1_ corresponds to an annual labor income of JPY 4.44 M, while *h*_2_ corresponds to that of JPY 6.84 M. The FP treatment eliminates benefit reductions in actuarial adjustments and moves Plan B to B^FP^. The CP treatment eliminates the current benefit suspension and moves Plan A to A^CP^. The FPCP treatment completely eliminates the ET and moves Plans A and B to A^CP^ and B^FP^, respectively. Labor income for Plans A and B is JPY 6.65 M (*h*_*H*_) and that of Plans C and D is JPY 4.44 M (*h*_*L*_).

Following previous studies, we use a one-period labor supply model to predict individual behavior for ET elimination [1, 5, 7, 8, 42]. This simple model illustrates the theoretical impact of ET elimination on labor supply decisions. Fig 1 shows workers’ budget constraints relating to the allocation between work and leisure, where the decision to claim pension is not considered for the time being. Without a public pension system, the budget constraint is FI (for reference), where F signifies total leisure (0% work hours). This line’s slope reflects the wage rate–*w*, omitting non-labor income, as per Japan’s earnings test. In a public pension system with an earnings test, we observe kinked budget constraints EJGI (our base case). No benefit is reduced if the work hours are less than the threshold *h*_*1*_ on segment JE. All benefits are taxed away beyond *h*_*2*_ on GI. The slope is–*w* (1–*τ*) for JG, where, mirroring Japan’s earnings test, the withholding (tax) rate *τ* is 50%; the threshold *h*_*1*_ and *h*_*2*_ set at annual incomes of JPY 4.44 M and JPY 6.84 M in our experimental setting, respectively.

Eliminating the ET shifts the budget constraints from EJGI to EK (our analytical target). The effects of this change differ depending on the initial location. Individuals initially on EJ face no change, while for JG, the impact is mixed: reduced labor supply by income effects and increased by substitution effects; the net effect remains an empirical question. Prior research has indicated that the substitution effect tends to prevail. For those on segment GI, eliminating the earnings test yields only negative income effects, suggesting less work. We recognize the limitation of this model—that is, labor adjustment may not be continuous and differ depending on family structure, spousal income, and financial asset holdings.

Next, the locations of each plan in our experiment are depicted in Fig 1. We initially assume that the pension-claiming age (65 or 66 years in our case) is indifferent because benefits are actuarially fairly adjusted. That is, the present values of benefits are equivalent on average regardless of claiming age because increased benefits in the future effectively neutralize forgone benefits through actuarial adjustments. We also assume no liquidity constraint, meaning life-contingent financial products can mimic delayed pension benefits, attaining the same cash flow even if the delayed pension claims are not available. Plan E is located where leisure is 100% (no work), and the individual receives pension benefits. Plans C and D are located on segment EJ (just right of J). Plans A and B are on JG (close to G). Labor income at age 65 in Plans C and D is JPY 4.43 M, corresponding to *h*_*L*_ in Fig 1. Similarly, labor income at age 65 in Plans A and B is JPY 6.65 M, corresponding to *h*_*H*_. As mentioned, we assume that the respondent is indifferent toward the choice of either Plans C or D (A or B). The ET elimination in the FPCP treatment moves Plans A and B to A^CP^ and B^FP^, respectively.

Finally, we consider individual variances in valuing actuarial adjustments. Respondents’ behavior varies because they may perceive actuarial adjustments as either favorable or unfavorable [7]. This variation is attributed to personal discount rates [43] and subjective life expectancy [44, 45]. Early or delayed claims decisions have long-term impacts on one’s life because benefits are fixed depending on the claiming age. We infer that respondents believing that actuarial adjustments favor them choose a plan with the delayed benefits (Plans B or D) and vice versa. Table 5 summarizes the factors affecting respondents’ plan choices based on Fig 1‘s theory and predictions from empirical studies according to our treatment setting.

**Table 5:** Factors affecting respondents’ choice according to treatment.

Treatment	Factors	Plan A	Plan B	Plan C	Plan D	Not choosing Plan E
		HIWP	HINP	LIWP	LINP	Work at 65
FP	Substitution effect		↑	↓	↓	
	Relative attractiveness	↓	↑			
	(Un)favorable actuarial adjustment	Yes				
	Labor market rigidity and fixed cost of labor participation					↑
	Empirical prediction for Eq (7)	*β*_1_ < 0	*β*_1_ > 0	*β*_1_ < 0	*β*_1_ < 0	*β*_1_ > 0
CP	Substitution effect	↑		↓	↓	
	Relative attractiveness	↑	↓			
	Favorable actuarial adjustment		Yes			
	Labor market rigidity and fixed cost of labor participation					↑
	Empirical prediction for Eq (7)	*β*_2_ > 0	*β*_2_ < 0	*β*_2_ < 0	*β*_2_ < 0	*β*_2_ > 0
FPCP	Substitution effect	↑	↑	↓	↓	
	Relative attractiveness					
	Favorable actuarial adjustment	Yes	Yes			
	Labor market rigidity and fixed cost of labor participation					↑
	Empirical prediction for Eq (7)	*β*_3_ > 0	*β*_3_ > 0	*β*_3_ < 0	*β*_3_ < 0	*β*_3_ > 0

**Note**: ↑ represents an increase in the probability of the outcome, while ↓ represents a decrease.

The CP treatment shifts Plan A to the A^CP^ located on JK in Fig 1, keeping Plan B at its original position. In the CP treatment (second row in Table 5), we expect respondents choosing Plan C or D in the first trial (control) to change to Plan A^CP^ by the substitution effect. In addition, respondents choosing Plan B in the first trial change to Plan A^CP^ because of its relative attractiveness. However, some respondents, considering longevity risk and believing that the actuarial adjustment is favorable to them, may remain in Plan B because it still offers increased benefits at age 66 or older (favorable actuarial adjustment).

Similarly, the FP treatment moves Plan B to B^FP^ on JK, keeping Plan A at its original position. We expect that the probability of choosing Plan B^FP^ will increase for those choosing Plans C or D in the first trial because of the substitution effect. In addition, some participants choosing Plan A in the first trial switch to Plan B^FP^ because of its relative attractiveness. However, some respondents, believing that the actuarial adjustment is unfavorable, remain in Plan A because it still offers the current benefits at age 65 (unfavorable actuarial adjustment). In the later section, we provide policy recommendations based on the respondents’ preferences, especially in the FP treatment, regarding increasing labor supply and delaying pension claims. Then, in the FPCP treatment, we expect that those choosing Plans C or D in the first trial change to Plans A^CP^ or B^FP^ by the substitution effect. Their choice depends on the subjective fairness of actuarial adjustment.

Note that, as our plans are discrete and respondents cannot adjust the labor supply continuously, we assume that respondents choose the closest plan for their labor supply preference. Strictly speaking, Plans C and D are on EJ and should not be affected by ET elimination. However, we assume Plans C and D are located around the kink. Empirical studies have shown that eliminating the ET increases the labor supply for those with incomes just below the kink point.

Although Plan E’s budget constraint remains unchanged in Fig 1‘s model, eliminating the ET could shift preferences within the FP, CP, and FPCP treatments owing to labor market dynamics. Individuals contending with labor market rigidity and the fixed costs of entering the labor market may initially opt for Plan E, as Baker and Benjamin [8] note. However, removing the ET might alleviate these barriers, potentially encouraging greater workforce participation in the FP, CP, and FPCP treatments, as predicted in the last column of Table 5 (Not choosing Plan E).

We recognize the difference between theoretical explanations and experimental settings. Our theory is conventionally explained sequentially, addressing labor supply and then pension claims. Respondents in our experiment may determine their plan in a different order. The theory outlined in this section is designed to simplify complex real-world phenomena, making them more comprehensible. However, to capture the nuances of real-world behavior more accurately, it will be important to consider more advanced theories in future research.

### Survey implementation

An Internet-based survey was conducted in Japan in February 2022, and respondents were contacted using the registered individuals at MyVoice.com Ltd (https://www.myvoice.co.jp). We conducted the two surveys for this study: preliminary and main surveys. The recruitment period for the preliminary survey was from February 4, 2022, to February 9, 2022, followed by the main survey from February 10, 2022, to February 15, 2022. This study has been approved by the review committee for research involving human participants at Konan University (21–19). All participants provided (computer-based) written informed consent. Recruitment targeted registered members of MyVoice.com, reached through Internet advertising. Upon registration, members provided demographic and socioeconomic information, including gender, age, place of residence, marital status, occupation, annual income, and family structure (https://voice.myvoice.co.jp/front/sign_up/top/). We randomly selected approximately 5,000 potential respondents for the preliminary survey using this information—this survey aimed to update their demographic and socioeconomic information, acknowledging potential changes since their initial registration. From those who completed the preliminary survey, male participants aged 40–59 years, married, employed by a company, and with a household income ranging from JPY 4.0 M to less than JPY 9.0 M were randomly chosen to participate in the main survey. We used household income instead of individual income to exclude extrema households such that the household head and spouse income are both JPY 9.0 M or more, which is almost a top-income household in Japan. Respondents were randomly allocated to the three treatment groups (Groups 1–3) to mitigate the effects of the treatment experience, as explained below. They were further divided into subgroups that were almost equally distributed in five-year age intervals (40–44, 45–49, 50–54, and 55–59) and high- and low-income groups: the low-income group comprises those whose household income is more than or equal to JPY 4.0 M and less than JPY 6.5 M, while the high-income comprises more than or equal to JPY 6.5 M and less than JPY 9.0 M. We created age subgroups, considering that the population compositions of these age ranges are almost the same. We also created income subgroups so that respondents’ actual annual incomes do not differ significantly from these experimental settings. Given these sub-groups, we ensured that the respondents’ age and annual income distribution were not highly skewed. We tried to collect approximately 90 respondents in each subgroup and 360 respondents in each group. Each respondent underwent four trials to choose Plans A–E.

Table 6 displays the treatment order according to the respondent group. Each group faced treatments in a different order to mitigate the experience of preceding decisions in measuring the overall average treatment effects. The first trial was the control (status quo) for all respondent groups. The treatment order from the second to fourth trials differed according to the group. For example, Group 1 respondents faced the FP treatment in the second trial and the CP and FPCP treatments in the third and fourth trials, respectively. Respondents answered the questions as presented in Tables 1–4, followed by questions about their personal attributes. They were rewarded with a fixed cash equivalent. Given that public pension benefits vary based on employees’ income and length of service, we limited respondents to certain occupations and annual income levels to align with our experimental setting. In addition, those with large heterogeneity in public pension benefits, such as self-employed individuals, were excluded.

**Table 6:** Order of treatment according to respondent group.

	Group 1	Group 2	Group 3
First trial	Control	Control	Control
Second trial	FP	CP	FPCP
Third trial	CP	FP	FP
Fourth trail	FPCP	FPCP	CP

**Note**: The FP treatment eliminates the reduction of actuarial adjustments. The CP treatment eliminates the current benefit suspension. The FPCP treatment completely eliminates the ET.

Respondents were informed about the purpose of the survey: the authors conducted this survey to empirically analyze the employment of older workers and the pension system at their research institutions. The study sought to analyze the pension system design and explore the requirements for a prosperous retirement. Respondents were also informed that the response data would be statistically analyzed and used to prepare materials, reports, and academic papers and no other purpose. To complete the survey, they were instructed to read the questions online and select the answers that best represented their situation. Therefore, survey participants responded knowing the survey was about pensions and employment. However, respondents were not explicitly told that the study was designed to examine reforms to the ET in the pension system. As explained in Tables 1–4, we did not use the term “retirement earnings test” in our survey. Instead, we specifically indicated labor income and pension amounts to overcome the limitation of respondents, who were mostly young and did not have specific knowledge of the ET. At the same time, more knowledgeable respondents may gain an erroneous impression of the ET and fail to consider the labor income and pension amounts presented properly. Instead, they choose answers indicating ET elimination. Presenting labor income and pension amounts as work plan options without specifically mentioning the ET would be more likely to elicit an honest preference for employment and pension benefits.

As a result, respondents more interested in old-age pensions and employment issues were more likely to participate in the survey. By contrast, those less interested may be less likely to participate. However, given Japan’s declining birthrate and aging population, interest in employment and pensions among middle and senior workers is high. We anticipate that the number of respondents who opt out of the survey will not be significant due to their disinterest in these topics. Nonetheless, we acknowledge that some sample bias related to interest in the survey persists, warranting caution in interpreting our findings and highlighting the need for further research.

### Empirical method

To estimate the treatment effects on senior workers’ employment and pension-claiming behaviors, we estimate the following regression:

$$Y_{i}=\beta_{0}+\beta_{1}FP_{i}+\beta_{2}CP_{i}+\beta_{3}FPCP_{i}+\gamma^{T}Z_{i}+\varepsilon_{i},$$ (7)

where *i* represents a respondent. The outcome variable *Y* represents the respondent’s choice of work plans, which takes six forms: HIWP, HINP, LIWP, LINP, High income, or Work65. HIWP, HINP, LIWP, and LINP equal 1 if a particular work plan is selected, and 0 otherwise. Furthermore, we use the dummies High income and Work65. High income equals 1 if a respondent chooses the high-income plan (HIWP or HINP) and 0 otherwise. Work65 equals 1 if a respondent chooses plans for work at 65 (Plans A–D) and 0 otherwise (Plan E: retire at 65).

The main independent variables are FP, CP, and FPCP, which capture the treatment settings. FP (enhancement of future pension) equals 1 if a respondent faces treatment FP and 0 otherwise. In the FP treatment, the ET for reducing future pension growth based on actuarial adjustments is eliminated, given that all other parameters are unchanged, as shown in Table 2. CP (enhancement of current pension) equals 1 if a respondent faces the CP treatment and 0 otherwise. In the CP treatment, the ET for suspending current benefits is eliminated, given that all other parameters are unchanged, as shown in Table 3. FPCP equals 1 if a respondent faces the FPCP treatment and 0 otherwise. In the FPCP treatment, the ET is completely eliminated, as shown in Table 4. Variable *Z* represents individual characteristics explained below. *β*_0_, *β*_1_, *β*_2_, *β*_3_, and *γ* are regression coefficients, and *ε*_*i*_ is the residual.

Our empirical analysis is structured as binary choice models for each of the four distinct work plans and two combinations of plans (High income and Work) that respondents may select, where the dependent variable *Y* in Eq (7) for each model represents the binary choice (0 or 1) associated with selecting a specific plan. Accordingly, we use the ordinary least squares (OLS) with pooled data. We conduct six separate regressions, each examining the impact of the three treatment conditions (FP, CP, and FPCP) on the likelihood of selecting each (combination of) plan (linear probability model). Each respondent experienced the control (status quo), FP, CP, and FPCP treatments once in different implementation orders in each group to which they belonged (the status quo was experienced first by all respondents). Because each plan reflects the characteristics of wages and pension benefits, we can directly assess the effectiveness of each treatment on respondents’ preferences for different wage and pension claiming plans.

Each regression estimates the average treatment effects on the binary outcomes. The coefficients *β*_1_, *β*_2_, and *β*_3_ capture these effects for the FP, CP, and FPCP treatments, respectively. Table 5 includes the empirical predictions according to the treatment. We expect that *β*_1_ < 0, *β*_2_ < 0, and *β*_3_ < 0 in the low-income plans (LIWP and LINP) for the FP, CP, and FPCP treatments, respectively, by the substitution effect. For high-income plans (HIWP and HINP), the signs of *β* depend on the treatment. For treatment FP, we expect *β*_1_ > 0 for HINP by the substitution effect and relative attractiveness, and *β*_1_ < 0 for HIWP by relative (un)attractiveness. For the CP treatment, we expect *β*_2_ > 0 for HIWP and *β*_2_ < 0 for HINP for the same reasons. For the FPCP treatment, we expect *β*_3_ > 0 for high-income plans and *β*_3_ < 0 for low-income plans due to the substitution effect.

We recognized that the repeated choice aspect of the experiment might generate transition dynamics in the respondents’ choices across different trials. However, the treatment order changes can mitigate these issues to analyze overall treatment effects. Our robustness check analysis also includes the results from the mixed logit model, which measures the treatment’s effect by directly using variables representing wage and pension benefits as explanatory variables with the repeated choice aspect. These results drive qualitatively the same conclusion as our main analysis, as explained later.

The individual characteristics variables (*Z*) used in the regression analysis are as follows: trust in the public pension, subjective survival probability, financial literacy, subjective health condition, university graduation status, status of working in small companies, mandatory retirement age of 65 years or older, household income, financial assets, house ownership, and age. Trust in the public pension is measured as the respondent’s assessment of their trust in the public pension on a scale of one (almost unreliable) to six (quite reliable). Agnew et al. [46] find that defined contribution pension plan participants who do not trust financial institutions are more likely to quit automatic enrollment plans. We expect respondents with higher trust in the public pension to delay their pension benefit-claiming age. Subjective survival probability is measured as a respondent’s assessment of their probability of survival until the age of 85. Respondents chose options ranging from less than 5%, 5%, ⋯, 95%, and more than 95% in increments of 5%. We coded the choice of less than 5% as 2.5% and more than 95% as 97.5% for the survival probability variable. The literature shows mixed results regarding the survival probability of retirement-related decision-making. Payne et al. [47] find that subjective life expectancies are related to expressed preferences for hedging longevity risks. By contrast, Brown [48] shows that it has an insignificant effect on reducing longevity risk.

Financial literacy is measured as the average score of three standard financial literacy quizzes that test the (a) respondents’ understanding of compound interest rates, (b) the relationship between the value of foreign investments and exchange rates, and (c) the diversification effect. Regarding (b), instead of inflation, we use foreign investment, following Lusardi and Mitchell [49], who state that the relationship between the quiz and people’s daily financial decisions is essential. In Japan, the exchange rate is related to daily consumption, such as food and energy, and foreign investment is more aligned with this concept than the inflation rate. Financial literacy is the average of the correct answer rates for the three quizzes. Higher scores indicate higher financial literacy, which affects individuals’ retirement planning and implementation [50]. The literature shows mixed results for financial literacy regarding retirement and public pension-claiming decisions. Brown et al. [51] find that individuals with low financial literacy are less likely to value annuities, or public pensions in our study. They might not fully understand the benefits of delaying pension claims due to actuarial adjustments. By contrast, Agnew et al. [52] find that individuals with high financial literacy are less likely to depend on annuities and are more likely to choose self-management. Such individuals tend to be more confident about stock and mutual fund investments and prefer to receive public pensions at the NPA. Household income is the sum of the respondent’s and spouse’s incomes, obtained through multiple-choice questions with 14 choices. Financial assets are also obtained through a multiple-choice question with 22 choices, including bank savings, stocks, mutual funds, and money market funds, excluding the house. Income and financial asset choices are presented in ranges, and the variables use their medians.

## Results

### Characteristics of respondents

Table 7 presents the descriptive statistics of the respondents’ characteristics. The trust for public pension is 3.0, which is neutral on average. The average survival probability at age 85 is 32.5%, far lower than the actual (objective) probability (42.5%) based on an average respondent age of 50 years. The average health condition is 4.3, which is relatively good. Table A1 in S1 Appendix presents the detailed responses for those categorical variables. The average correct answer rate for the financial literacy quizzes is 59.0%, which is within the range of results in the literature. Household income and financial assets are JPY 7.0 M and 8.9 M, respectively. Our respondents’ higher income and financial asset levels, compared to the national average, may be attributed to our targeted screening process during recruitment. The average income of respondents is JPY 5.8 M, which is on the segment GJ in Fig 1. We believe this income level seems appropriate for choosing between a high- or low-income plan at age 65. Table A2 in S1 Appendix presents the differences in individual characteristics between our sample and a large-scale governmental survey for the selected variables. The characteristics of our respondents align closely with those from the larger survey, except for education levels, a variation that could be attributed to the online nature of our survey. Table A3 in S1 Appendix presents the individual characteristics by respondent group. While minor differences are noted, we believe they do not significantly influence our analysis.

**Table 7:** Descriptive statistics of respondents.

Variable	Unit	Mean	Std.	Min	Max
Trust for public pension	Scale 1 (Almost unreliable)–6 (Quite reliable)	3.04	1.27	1	6
Survival probability at age 85	%	32.51	26.88	2.5	97.5
Financial literacy		0.59	0.37	0	1
Health condition	Scale 1 (Fairly poor)-6 (Fairly good)	4.25	1.08	1	6
University	(d)	0.75	0.43	0	1
Small company	(d)	0.50	0.50	0	1
Mandatory retirement age 65 or more	(d)	0.34	0.48	0	1
Household income	JPY Million	6.97	1.74	3.05	12
Respondent’s income	JPY Million	5.80	1.61	2.15	12
Financial assets	JPY Million	8.92	17.60	0	150
House own without mortgage	(d)	0.25	0.43	0	1
House own with mortgage	(d)	0.49	0.50	0	1
House rent	(d)	0.27	0.44	0	1
Age	Years old	49.98	5.55	40	59
N		1,061			

**Note**: (d) represents a dummy variable.

### Transition probability by treatment effect

This subsection analyzes the respondents’ choices during the first and second trials to capture their initial behavior through straightforward analysis, as explained in Table 5. Each respondent’s first trial represents the control condition (status quo), and the second trial introduces their first experience with a treatment condition, varying according to the respondent’s group as outlined in Table 6. This setup ensures consistent comparison conditions across treatments. However, for the third and fourth trials, respondents encounter treatments based on their group allocation, introducing variability due to the experience effect, where prior exposure to treatments may influence subsequent responses. This variability presents challenges for direct comparison of later trials. We will explore the results of regression analyses using all data in the subsequent subsections, aiming to provide comprehensive insights into the treatment effects.

Table 8 displays the transition probabilities of the plan choices from the first to second trials according to the treatments. Panel A shows the transition probabilities of the FP treatment (Group 1). In the first trial (control), 92% of respondents chose to work at age 65 (8% did not work). The highest probability of being selected is HINP (29%), and the other working plans are almost similar (20–22%). In the second trial, the FP treatment increases the pension benefits at age 66 in HINP (Plan B). The probability of changing to HINP from low-income plans is high: 57% (32%) of respondents who select LINP (LIWP) in the first trial change to HINP in the second trial, which is consistent with the substitution effect. Among the high-income plans, approximately half of the respondents who select HIWP in the first trial change to HINP, which is consistent with the relative attractiveness. The remaining half maintains HIWP, consistent with the unfavorable actuarial adjustment. These results are consistent with the predictions presented in Table 5.

**Table 8:** Transition probabilities by treatment.

**Panel A. FP treatment (Group 1, N = 352).**									
		Freq. of		Second trial					
		first trial		Plan A	Plan B	Plan C	Plan D	Plan E	Total
		N	Freq.	HIWP	HINP	LIWP	LINP	No Work	
First trial	HIWP	71	20%	51%	48%	1%	0%	0%	100%
	HINP	101	29%	0%	91%	3%	5%	1%	100%
	LIWP	77	22%	3%	32%	60%	5%	0%	100%
	LINP	75	21%	0%	57%	7%	36%	0%	100%
	No Work	28	8%	0%	11%	0%	7%	82%	100%
	Total	352	100%	11%	56%	16%	11%	7%	100%
**Panel B. CP treatment (Group 2, N = 354).**									
		Freq. of		Second trial					
		first trial		Plan A	Plan B	Plan C	Plan D	Plan E	Total
		N	Freq.	HIWP	HINP	LIWP	LINP	No Work	
First trial	HIWP	71	20%	93%	4%	3%	0%	0%	100%
	HINP	92	26%	48%	45%	4%	3%	0%	100%
	LIWP	82	23%	43%	1%	52%	4%	0%	100%
	LINP	78	22%	17%	5%	12%	65%	1%	100%
	No Work	31	9%	6%	0%	6%	16%	71%	100%
	Total	354	100%	45%	14%	17%	18%	7%	100%
**Panel C. FPCP treatment (Group 3, N = 355)**									
		Freq. of		Second trial					
		first trial		Plan A	Plan B	Plan C	Plan D	Plan E	Total
		N	Freq.	HIWP	HINP	LIWP	LINP	No Work	
First trial	HIWP	64	18%	81%	19%	0%	0%	0%	100%
	HINP	102	29%	16%	80%	3%	1%	0%	100%
	LIWP	83	23%	23%	16%	59%	0%	2%	100%
	LINP	82	23%	5%	49%	5%	40%	1%	100%
	No Work	24	7%	8%	4%	0%	0%	88%	100%
	Total	355	100%	26%	42%	16%	10%	7%	100%

**Note:** Each table represents the frequency of the first trial and the transition probabilities according to treatments. The rows show the respondents’ selection in the first trial and the columns show their selections in the second trial. The samples are used only in the first and second trials.

Panel B shows the transition probabilities for the CP treatment (Group 2). The frequency of choosing each plan at the first trial is similar to that in Panel A. HINP has the highest probability of being selected (26%). The transition probabilities also have a similar tendency to those in Panel A. For the second trial, the CP treatment increases the pension benefits at age 65 in HIWP (Plan A). The probability of changing from low-income plans (LIWP and LINP) to HIWP is high (43% and 17%, respectively), consistent with the substitution effect. Approximately half of the respondents who select HINP change to HIWP, consistent with the relative attractiveness. The remaining half maintains HINP, consistent with the favorable actuarial adjustment.

Panel C shows the transition probabilities for the FPCP treatment (Group 3). For the first trial, the frequency of choosing each plan is similar to that in Panel A. For the second trial, the FPCP treatment eliminates the ET completely and increases pension benefits at age 66 or older in HINP and age 65 in HIWP. The probabilities of changing from low-income to high-income plans are high (5–49%), consistent with the substitution effect. The respondents exhibit a specific tendency in their choices: Those who choose LINP in the first trial tend to change to HINP (similarly, LIWP to HIWP). After the complete elimination of the ET, the only difference between HIWP and HINP is the timing of the claiming age for actuarially fair benefits. Therefore, the preferences for these plans should theoretically be indifferent. However, respondents’ pension benefits-claiming behavior is strongly related to the subjective evaluation of actuarial adjustments even under ET elimination, consistent with Disney and Smith [7]. For example, the sum of the transition probabilities from LIWP to plans that include pension claiming at age 65 (HIWP and LIWP) is 82%. Similarly, the sum of the transition probabilities from LINP to plans that include pension claiming at age 66 (HINP and LINP) is 89%.

### Main results

Table 9 presents the estimation results based on Eq (7). The outcome is an indicator that represents the work plan and its combinations. The primary independent variables are FP, CP, and FPCP, which capture the extent of elimination of the ET. FP eliminates the reduction in actuarial adjustments, and CP removes the suspension of current pension benefits. FPCP eliminates both the reduction in actuarial adjustments and suspension of benefits. We employ a linear probability model to estimate Eq (7), with standard errors clustered at the respondent level.

**Table 9:** Effect of eliminating earnings test.

Outcome	(1)		(2)		(3)		(4)		(5)		(6)	
	HIWP		HINP		LIWP		LINP		High income		Work65	
FP	-0.044	***	0.230	***	-0.049	***	-0.132	***	0.186	***	0.005	
(Enhance future pension)	(0.011)		(0.016)		(0.011)		(0.012)		(0.014)		(0.005)	
CP	0.237	***	-0.094	***	-0.071	***	-0.059	***	0.142	***	0.012	***
(Enhance current pension)	(0.015)		(0.014)		(0.012)		(0.010)		(0.013)		(0.005)	
FPCP	0.105	***	0.117	***	-0.076	***	-0.132	***	0.221	***	0.013	***
(Complete elimination of ET)	(0.013)		(0.016)		(0.012)		(0.012)		(0.015)		(0.005)	
Trust in public pension	-0.012		0.033	***	-0.009		0.003		0.022	**	0.016	***
	(0.008)		(0.009)		(0.007)		(0.006)		(0.009)		(0.006)	
Survival probability at age 85	-0.000		0.001	**	-0.001	***	0.001	*	0.001		0.000	
	(0.000)		(0.000)		(0.000)		(0.000)		(0.000)		(0.000)	
Financial literacy	0.032		-0.036		-0.030		0.037		-0.004		0.003	
	(0.030)		(0.030)		(0.028)		(0.023)		(0.035)		(0.021)	
Health condition	0.009		-0.001		0.006		-0.003		0.008		0.011	*
	(0.010)		(0.010)		(0.009)		(0.007)		(0.012)		(0.007)	
University	-0.038		0.057	**	0.003		-0.036		0.019		-0.014	
	(0.025)		(0.025)		(0.023)		(0.022)		(0.029)		(0.017)	
Small company	-0.006		-0.005		-0.011		0.026		-0.012		0.003	
	(0.021)		(0.022)		(0.019)		(0.018)		(0.025)		(0.015)	
Mandatory retirement age at 65 or more	-0.005		0.020		-0.006		0.007		0.016		0.016	
	(0.022)		(0.022)		(0.020)		(0.018)		(0.026)		(0.015)	
Household income	0.011	*	0.009		-0.006		-0.005		0.020	***	0.009	*
	(0.006)		(0.006)		(0.006)		(0.005)		(0.007)		(0.005)	
Financial assets	-0.001		-0.002	***	0.000		0.001		-0.002	***	-0.001	**
	(0.001)		(0.001)		(0.001)		(0.001)		(0.001)		(0.001)	
Own house while paying mortgage loan	0.033		-0.003		0.018		-0.003		0.030		0.044	**
	(0.027)		(0.027)		(0.026)		(0.022)		(0.032)		(0.020)	
House rent	-0.009		0.076	**	-0.008		-0.011		0.066	*	0.048	**
	(0.028)		(0.030)		(0.027)		(0.024)		(0.035)		(0.023)	
Age	-0.006	***	-0.001		-0.000		0.003	*	-0.007	***	-0.005	***
	(0.002)		(0.002)		(0.002)		(0.002)		(0.002)		(0.001)	
Constant	0.454	***	0.103		0.315	***	0.098		0.557	***	0.970	***
	(0.129)		(0.128)		(0.115)		(0.092)		(0.145)		(0.081)	
N	4,244		4,244		4,244		4,244		4,244		4,244	
F-test	28.60	***	35.54	***	3.87	***	10.08	***	19.66	***	3.21	***

**Note**: This table presents the estimation results based on Eq (7). The first row represents the outcome variables: high income with pension benefits (Plan A: HIWP), high income without pension benefits (Plan B: HINP), low income with pension benefits (Plan C: LIWP), low income without pension benefits (Plan D: LINP), high income (HIWP or HINP), and Work65 (HIWP, HINP, LIWP, or LINP). FP is an indicator of the FP treatment that eliminates the reductions of actuarial adjustments. CP is an indicator of CP treatment that eliminates the current benefit suspension. FPCP is an indicator of the treatment of FPCP, which completely eliminates the ET. Standard errors clustered at the respondent level are in parentheses.

***, **, and * indicate statistical significance at *p* < 0.01, *p* < 0.05, and *p* < 0.1, respectively.

Regarding the intensive margin (change in labor income), the FP treatment significantly decreases the choice of low-income plans and increases the choice of HINP. The FP treatment reduces the probability of choosing LIWP by 4.9 percentage points in Column (3) and 13.2 percentage points for LINP in Column (4). It also increases the probability of choosing HINP by 23.0 percentage points in Column (2), which is consistent with the substitution effect. In Column (1), the FP treatment reduces the probability of choosing HIWP by 4.4 percentage points, consistent with its relative (un)attractiveness among high-income plans. Some respondents remain in the HIWP, as actuarial adjustments are unfavorable for them, and prefer a plan to receive pension benefits at age 65. The predicted probability of HIWP evaluated at FP = 0, CP = 0, and FPCP = 0 (control) is 19.4%, whereas that at FP = 1, CP = 0, and FPCP = 0 (treatment FP) is 15.0%. Indeed, ET reform accelerates earlier benefit claims [3]. By contrast, the FP treatment encourages labor supply and delayed benefit claims, which is a novel finding. In addition, those with higher trust in public pensions and a higher survival probability at age 85 tend to choose HINP. These respondents may be concerned about their longevity risk.

Second, the CP treatment significantly decreases the choice of low-income plans and increases the choice of HIWP. It reduces the probability of choosing LIWP by 7.1 percentage points in Column (3) and 5.9 percentage points for LINP in Column (4). It increases the probability of choosing HIWP by 23.7 percentage points in Column (1), which is consistent with the substitution effect. In Column (2), the CP treatment reduces the probability of choosing HINP by 9.4 percentage points, indicating relative (un)attractiveness. Some respondents remain in HINP, given favorable actuarial adjustments, and prefer a plan with delayed benefits. The predicted probability of HINP evaluated at FP = 0, CP = 0, and FPCP = 0 is 27.8%, whereas that of FP = 0, CP = 1, and FPCP = 0 (treatment CP) is 18.4%. Table A4 in S1 Appendix presents the results segmented by respondent group (Panel A). Notable differences are observed in the FP and CP treatments across groups, indicating the presence of experience effects (Panel B). Varying treatment sequences mitigate this concern.

Third, the FPCP treatment significantly decreases the choice of low-income plans and increases the choice of high-income plans. It reduces the probability of choosing LIWP by 7.6 percentage points in Column (3) and 13.2 percentage points for LINP in Column (4). It increases HIWP by 10.5 percentage points in Column (1) and HINP by 11.7 percentage points in Column (2), consistent with the substitution effect. In Column (2), the FPCP treatment weakly increases HINP (–0.113 = 0.117–0.230) compared with the FP treatment (*p* < 0.01) because the FP treatment includes relative attractiveness in addition to substitution effects.

Fourth, we observe distinct sensitivities among respondents to the different treatments. The FP treatment reduces the likelihood of selecting LINP by 13.2%, a more pronounced effect than the LIWP plan, which decreases by 4.9%, indicating a greater sensitivity among respondents opting for delayed benefits to the FP treatment. Conversely, the CP treatment shows a stronger reduction in preference for LIWP (–7.1%) over LINP (–5.9%) and a notable increase in preference for the HIWP, underscoring the sensitivity of respondents favoring immediate benefits towards CP. Similarly, the FPCP treatment exhibits a more significant decrease in the preference for LINP (–13.2%) compared to LIWP (–7.6%) and a larger increase in preference for HINP (+11.7%) over HIWP (+10.5%). These patterns suggest that decisions regarding pension benefit claims under the ET elimination are influenced by the perceived fairness of actuarial adjustments, aligning with the findings of Disney and Smith [7].

Finally, in Column (5), the FP, CP, and FPCP treatments significantly increase the choice of high-income plans. The effect of FP exceeds that of CP; the difference in coefficients is 0.043 (*p* < 0.01), indicating that the FP treatment is more likely to be a potential policy candidate for ET reform.

Next, regarding the extensive margin (work or not), Column (6) shows that the CP and FPCP treatments significantly increase Work65, which the labor supply model in Fig 1 does not predict. The result indicates that some respondents who choose not to work at age 65 (Plan E) may be subject to the fixed costs of work that prevent workers from freely choosing work hours. Eliminating the ET relaxes this constraint, as Baker and Benjamin [8] note. Contrary to these treatments, FP alone is insignificant for work, which aligns with the unfavorable actuarial adjustment. The respondents who choose not to work at age 65 and receive pension benefits do not prefer to delay the claiming age, even though the FP increases the delayed benefits.

Regarding the control variables, more financial assets decrease the choice of HINP, High income, and Work, indicating that those with sufficient financial assets are less willing to work. Financial literacy has an insignificant effect on the choice of work plans. Basic financial literacy may not impact the deferments of public pension benefits, and other skills, such as numeracy and knowledge of financial products, may be required to understand the benefits of delaying public pension claims [53]. Note that we estimate other specifications, such as excluding the FPCP treatment dummy variable, to check the robustness of our results. Nevertheless, our conclusions remain qualitatively the same.

### Robustness checks

#### Analysis of data divided into subsamples

We estimate Eq (7) for three subsample categories: household income, financial assets, and age. The effect of ET elimination may differ by the respondents’ income subgroups. Research shows that this effect is expected to be more significant for respondents just below the threshold [5, 9]. Panels A and B in Table 10 present the results regarding whether the household income subsample is lower and higher than the median. We report only the coefficients of FP, CP, and FPCP, and omit the coefficients of the control variables. The overall results in Panels A and B are similar to those in Table 9. FP (CP) increases HINP (HIWP) and decreases low-income plans. The FPCP increases and decreases high- and low-income plans, respectively. The magnitudes of these changes are similar in Panels A and B.

**Table 10:** Treatment effects by subsample.

**Panel A. Low income (N = 2,456).**												
Outcome	HIWP		HINP		LIWP		LINP		High income		Work	
FP	-0.044	***	0.238	***	-0.047	***	-0.143	***	0.194	***	0.003	
	(0.013)		(0.021)		(0.015)		(0.016)		(0.019)		(0.007)	
CP	0.235	***	-0.096	***	-0.072	***	-0.059	***	0.138	***	0.008	
	(0.019)		(0.018)		(0.017)		(0.014)		(0.018)		(0.007)	
FPCP	0.109	***	0.119	***	-0.078	***	-0.138	***	0.228	***	0.011	*
	(0.017)		(0.020)		(0.017)		(0.015)		(0.020)		(0.006)	
**Panel B. High income (N = 1,788).**												
Outcome	HIWP		HINP		LIWP		LINP		High income		Work	
FP	-0.045	**	0.219	***	-0.051	***	-0.116	***	0.174	***	0.007	
	(0.018)		(0.024)		(0.017)		(0.018)		(0.021)		(0.006)	
CP	0.239	***	-0.092	***	-0.069	***	-0.060	***	0.148	***	0.018	***
	(0.023)		(0.023)		(0.018)		(0.015)		(0.020)		(0.006)	
FPCP	0.098	***	0.114	***	-0.074	***	-0.123	***	0.213	***	0.016	**
	(0.022)		(0.026)		(0.018)		(0.017)		(0.022)		(0.007)	
**Panel C. Low financial assets (N = 2,292).**												
Outcome	HIWP		HINP		LIWP		LINP		High income		Work	
FP	-0.040	***	0.229	***	-0.047	***	-0.140	***	0.188	***	0.002	
	(0.015)		(0.022)		(0.015)		(0.017)		(0.020)		(0.006)	
CP	0.229	***	-0.080	***	-0.068	***	-0.073	***	0.148	***	0.007	
	(0.020)		(0.019)		(0.017)		(0.014)		(0.019)		(0.006)	
FPCP	0.091	***	0.133	***	-0.066	***	-0.147	***	0.223	***	0.010	*
	(0.018)		(0.021)		(0.016)		(0.016)		(0.019)		(0.006)	
**Panel D. High financial assets (N = 1,952).**												
Outcome	HIWP		HINP		LIWP		LINP		High income		Work	
FP	-0.049	***	0.232	***	-0.051	***	-0.123	***	0.182	***	0.008	
	(0.015)		(0.023)		(0.017)		(0.017)		(0.020)		(0.007)	
CP	0.246	***	-0.111	***	-0.074	***	-0.043	***	0.135	***	0.018	**
	(0.022)		(0.021)		(0.018)		(0.015)		(0.020)		(0.007)	
FPCP	0.121	***	0.098	***	-0.088	***	-0.115	***	0.219	***	0.016	**
	(0.020)		(0.024)		(0.018)		(0.017)		(0.022)		(0.008)	
**Panel E. Age 40s (N = 2,124).**												
Outcome	HIWP		HINP		LIWP		LINP		High income		Work	
FP	-0.047	***	0.241	***	-0.056	***	-0.126	***	0.194	***	0.011	**
	(0.017)		(0.024)		(0.017)		(0.016)		(0.020)		(0.005)	
CP	0.260	***	-0.115	***	-0.077	***	-0.053	***	0.145	***	0.015	**
	(0.022)		(0.021)		(0.018)		(0.015)		(0.021)		(0.006)	
FPCP	0.124	***	0.102	***	-0.094	***	-0.115	***	0.226	***	0.017	***
	(0.021)		(0.024)		(0.018)		(0.016)		(0.021)		(0.006)	
**Panel F. Age 50s (N = 2,120).**												
Outcome	HIWP		HINP		LIWP		LINP		High income		Work	
FP	-0.042	***	0.219	***	-0.042	***	-0.138	***	0.177	***	-0.002	
	(0.013)		(0.021)		(0.016)		(0.018)		(0.020)		(0.007)	
CP	0.213	***	-0.074	***	-0.064	***	-0.066	***	0.140	***	0.009	
	(0.020)		(0.018)		(0.016)		(0.013)		(0.017)		(0.007)	
FPCP	0.085	***	0.132	***	-0.058	***	-0.149	***	0.217	***	0.009	
	(0.017)		(0.022)		(0.016)		(0.017)		(0.020)		(0.007)	

**Note**: Respondents in Panels A and B are limited to those with household income below and above its median, respectively. Panels C and D include respondents whose financial assets are below and above its median, respectively. Panels E and F include respondents in their 40s and 50s, respectively. FP is an indicator of the FP treatment that eliminates the reductions of actuarial adjustments. CP is an indicator of CP treatment that eliminates the current benefit suspension. FPCP is an indicator of the treatment of FPCP, which completely eliminates the ET. The coefficients of control variables are omitted. Standard errors clustered at the respondent level are in parentheses.

***, **, and * indicate statistical significance at *p* < 0.01, *p* < 0.05, and *p* < 0.1, respectively.

Next, holding sufficient financial assets can contribute to lowering longevity risk. We predict that respondents’ financial assets can affect their decisions. Panels C and D show the results for the subsamples wherein respondents’ financial assets are lower and higher than the median, respectively. Again, the overall results in Panels C and D are similar to those in Table 9.

Finally, we also predict that respondents’ age (the decision span until retirement) affects their decisions because of the high correlation between age and income. Panels E and F show the results for the subsamples with respondents in their 40s and 50s, respectively. Again, the overall results in Panels E and F are similar to those in Table 9.

In sum, these results indicate limited effects of household income, financial assets, and age differences on ET elimination. The results could be attributed to a relatively homogenous sample. While this may be desirable in our study, how respondents with other characteristics would respond to the ET reform remains to be explored.

#### Analysis using multinomial logit model

To check the robustness of our results, we estimate a multinomial logit model, where the outcome is a dummy that represents a particular plan (Plans A–E), and the independent variables are the same as our main specification in Eq (7). Table 11 presents the marginal effect of the multinomial logit model. These results are similar to those presented in Table 9 and yield qualitatively the same conclusions.

**Table 11:** Result based on multinominal logit model (marginal effects).

Dependent variable	(1)		(2)		(3)		(4)		(5)	
	HIWP		HINP		LIWP		LINP		No Work	
	(Plan A)		(Plan B)		(Plan C)		(Plan D)		(Plan E)	
FP	-0.044	***	0.230	***	-0.049	***	-0.132	***	-0.005	
	(0.011)		(0.016)		(0.011)		(0.012)		(0.005)	
CP	0.237	***	-0.094	***	-0.071	***	-0.059	***	-0.012	***
	(0.015)		(0.014)		(0.012)		(0.010)		(0.005)	
FPCP	0.105	***	0.117	***	-0.076	***	-0.132	***	-0.013	***
	(0.013)		(0.016)		(0.012)		(0.011)		(0.005)	
Control variable	Yes		Yes		Yes		Yes		Yes	
N	4,244									
chi2	744.2									

**Note**: The outcome is either Plan A (HIWP), Plan B (HINP), Plan C (LIWP), Plan D (LINP), or Plan E (no work). Standard errors clustered at the respondent level are in parentheses. The FP treatment eliminates the reductions in actuarial adjustments. The CP treatment eliminates the current benefit suspension. The FPCP treatment completely eliminates the ET. The table shows the marginal effects. Control variables are included, but not displayed.

***, **, and * indicate statistical significance at *p* < 0.01, *p* < 0.05, and *p* < 0.1, respectively.

#### Analysis using mixed logit model

For our main analysis, we used treatment dummy variables to measure the treatment effects directly. We now examine the respondents’ wage and pension benefits preferences according to our treatment settings. Because a discrete choice experiment inspires our experimental settings, we employ a mixed logit model to evaluate respondent preferences, which can handle the transition dynamics of respondents’ choices. An analysis using a mixed logit model includes attribution (e.g., labor income and pension benefits) and levels (their values). As in our experiment, assuming labor income and benefits are received at different times (at ages 65 and 66 or older) complicates the direct application of these variables. In addition, a discrete choice experiment typically utilizes trade-offs, such as giving up a higher price to obtain better quality to infer the respondent’s preference. However, respondents tend to prefer higher labor income and pension benefits with no apparent trade-offs in our settings. Therefore, we use the reduction of labor income relative to high income (JPY 6.65 M) for our price attribute, which is defined as JPY 6.65 M–each plan’s labor income. We also introduce the concept of social security wealth (SSW), which is the present value of pension benefit streams, given survival for our non-price attribute, which can handle the difference in benefit receiving timings. The survival probability is obtained from the 23rd Life Table (Male). We assume the discount rate to be 4.88% for this section, such that the SSW of Plan C is the same as that of Plan D. We also use a dummy for delaying the age of pension claims to 66 years as well as that of work. Table 12 summarizes these attributes and levels for each life plan.

**Table 12:** Attributes and levels for mixed logit analysis.

Treatment	Plan A	Plan B	Plan C	Plan D	Plan E	Plan A	Plan B
	Control	Control	Control	Control	Control	CP FPCP	FP FPCP
Reduction of labor income	0	0	222	222	665	0	0
Social security wealth (SSW)	2,394	2,387	2,504	2,504	2,504	2,504	2,504
Delay claims (d)	0	1	0	1	0	0	1
Work at 65 (d)	1	1	1	1	0	1	1
For reference							
Labor income at 65	665	665	443	443	0	665	665
Pension benefits at 65	88	0	198	0	198	198	0
Pension benefits at 66 or older	198	205	198	215	198	198	215

**Note:** All values are in annual JPY (10,000), except for (d), which is a dummy variable. The reduction in labor income is calculated as 665 minus the labor income for each plan. SSW represents the present value of benefit streams, assuming survival, with a discount rate of 4.88%. For reference, labor income at age 65, pension benefits at ages 65, and 66 or older are also provided.

The conditional logit model is conventionally used to analyze choice experiment data [54]. Because its underlying assumption (i.e., independence of irrelevant alternatives [IIA]) is considered unrealistic and generally does not hold, Train [55] propose a less restrictive mixed logit model (MLM), also called a random parameter logit model, that allows for heterogeneity of individual preferences and a relaxation of the IIA assumption. In the MLM, some parameters are specified as random, implying that each respondent has a different parameter set. The outcome is the respondent’s choice of work plan (Choice), either 0 (not chosen) or 1 (chosen). The primary independent variables are the reduction of labor income, SSW, and the interaction term of SSW and treatment (SSW×FP and SSW×CP). We assume that the SSW coefficient follows a normal distribution. The variable FP for this section is an indicator that takes a value of 1 for FP and FPCP treatments, including the plan to eliminate the reduction of actuarial adjustment; it is 0 otherwise. The variable CP is also an indicator that takes a value of 1 for CP and FPCP treatments, including the plan to eliminate the suspension of current labor income; it is 0 otherwise. We use these variables because the SSWs have no variations among work plans in the FPCP treatment, and the MLM cannot be estimated with the FPCP indicator. We contend that these FP and CP indicators still capture the difference between the FP and CP treatments, although both include the FPCP treatment.

Table 13 displays the results using the MLM [56]. Column (1) displays the estimated coefficients and standard errors; Column (2) displays the willingness to pay (WTP), or subjective valuation, for each attribute: a marginal value of the attribute relative to reduced labor income (the price attribute). The coefficients of the reduction of labor income are negative, indicating that a plan with a higher reduction of labor income is less likely to be chosen. By contrast, those of SSWs are positive, indicating that a plan with a higher SSW is likely to be chosen. These results are consistent with predicted preferences of respondents. The coefficients of SSW×FP and SSW×CP are positive, meaning that a plan with higher SSW is more likely to be chosen compared with the status quo in the FP and CP treatments. In Column (2), the subjective valuations in the FP and CP treatments are 2.115 and 1.209, respectively, indicating that respondents value SSW, or pension benefits, relative to losing current income compared with the status quo. The difference in these subjective valuations confirms that respondents value pension benefits more in the FP treatment than in the CP treatment. These results are consistent with our main analysis. Column (3) adds indicators that delay claims and work at age 65, and the results are similar to that in Column (1). The coefficient of work at age 65 is negative, indicating that a plan involving work at age 65 is less likely to be chosen after considering the pension benefits under SSW. The lower part of the panel reveals that the estimated standard deviations for SSW are statistically different from zero, reflecting unobserved heterogeneities among our respondents.

**Table 13:** Result of mixed logit analysis.

Outcome	(1)		(2)		(3)		(4)	
	Choice				Choice			
	Mixed logit model		Subjective valuation (WTP)		Mixed logit model		Subjective valuation (WTP)	
	Coeff.		EST		Coeff.		EST	
	(SE)		(SE)		(SE)		(SE)	
**Mean**								
Reduction of labor income (Price attribute)	-0.003	***			-0.005	***		
	(0.000)				(0.000)			
Social security wealth (SSW)	0.007	***	2.037	***	0.009	***	1.858	***
	(0.001)		(0.220)		(0.001)		(0.149)	
SSW×FP treatment (F)	0.007	***	2.115	***	0.005	***	1.013	***
	(0.001)		(0.398)		(0.001)		(0.236)	
SSW×CP treatment (C)	0.004	***	1.209	***	0.003	***	0.583	***
	(0.001)		(0.349)		(0.001)		(0.209)	
Delay claims					0.063		12.692	
					(0.056)		(11.142)	
Work at 65					-1.411	***	-284.725	***
					(0.190)		(28.312)	
Difference [= (F)–(C)]			0.906	**			0.430	*
			(0.427)				(0.259)	
**Standard deviation**								
Social security wealth (SSW)	0.011	***			0.011	***		
	(0.001)				(0.001)			
N	21,220				21,220			
Chi-square	442.7				1,092.5			

**Note**: Columns (1) and (3) present the results from the mixed logit model, with standard errors (clustered at the respondent level) shown in parentheses. The Choice variable represents the outcome, taking a value of 0 (not chosen) or 1 (chosen) for each work plan. The SSW variable, representing the present value of pension benefit streams assuming survival, is assumed to follow a normal distribution in its coefficient. Columns (2) and (4) reveal the corresponding willingness to pay (WTP), with standard errors calculated using the delta method.

***, **, and * indicate statistical significance at *p* < 0.01, *p* < 0.05, and *p* < 0.1, respectively.

## Discussion and policy implications

We find that labor supply increases by eliminating the ET, consistent with the literature. However, its magnitude differs. Shimizutani and Oshio [9] find that the low-income share declined by 2–6 percentage points after eliminating the ET in 1985 based on observational data. Using simulations, Oshio et al. [2] find that full-time work increases by +1.6 percentage points after eliminating the ET. Our results show that high-income work increases by approximately +20 percentage points (e.g., Column (5) of Table 9), greater than in these studies. While the exact causes are unclear, one factor may be that our study relies on the respondents’ hypothetical choices, where our experimental setting is discrete, and respondents cannot adjust their labor supply continuously. They may overreact to eliminating the ET. Therefore, caution is warranted while discussing the absolute level of the respondents’ choices.

Despite the above limitation, our findings have certain policy implications. Because of the declining birthrate and aging population in Japan, a labor shortage and worsening of the financial condition of public pensions are expected. As explained, the NPA has increased to age 65, and senior worker employment has been promoted. If the policy goal is to enhance high-income work for highly skilled workers and delay the public pension-claiming age to hedge longevity risk, eliminating the reduced actuarial adjustment while maintaining the suspension in current pension benefits can be a policy worth considering. It also benefits public pension finance because individuals contribute premiums while working.

## Conclusion

This study uses hypothetical survey experiments to examine how eliminating the ET rule for those aged 65 years or older in Japan affects the male labor supply and public pension benefit-claiming behavior. This study focused on the behaviors of those currently working (aged 40–59 years) and expected to face an ET during their retirement. We consider extensive (working or not) and intensive margins (changes in labor income). The results show that eliminating the ET can promote labor supply after the NPA. Notably, the treatments with different patterns of the ET have heterogeneous effects. The current ET in Japan reduces immediate benefits and future pension growth if an individual earns more than the predetermined threshold. Eliminating the reduction in actuarial adjustment tends to increase high-income work without increasing early public pension claims. By contrast, eliminating suspended current pension benefits tends to increase high-income work and early pension benefit claims. The results of this study are consistent with the argument that the choice to defer pension benefits depends on the subjective evaluation of actuarial adjustment. Workers planning to delay their public pension age are more sensitive to delaying their decision to claim benefits if the reduction of actuarial adjustment is eliminated.

This study has some limitations. First, because of the hypothetical nature of the experimental setting, respondents could freely choose their work status and income without incurring an additional burden. Therefore, being aware of the limitations while interpreting the results gives more clarity to this study. Second, we only examine responses of men with an income around the ET threshold, where one may expect to find the greatest increase in labor supply if the ET is eliminated. Other groups, such as women and those outside our sample income range, may exhibit different responses. Furthermore, retirement may be a joint decision with a spouse, not incorporated into our experimental setting. These issues should be addressed in future studies.

## Supporting information

S1 File (ZIP)

S1 Appendix (DOCX)

## Abbreviations

BP: Basic pension
EPI: Employees’ pension insurance
ET: Earnings test
HINP: High income without pension benefits
HIWP: High income with pension benefits
JPY: Japanese Yen
LINP: Low income without pension benefits
LIWP: Low income with pension benefits
NPA: Normal pension age
