# Supplementary material for: Impact of eliminating retirement earnings test on labor supply and pension benefit claims
Source: PLoS One. 2024 Aug 9;19(8):e0304458. doi: 10.1371/journal.pone.0304458 (PMC11315313; doi:10.1371/journal.pone.0304458)
Supplement: S1 File — (ZIP) [file pone.0304458.s001.zip › Supporting Information/OnlineAppendix.pdf]

Impact of eliminating retirement earnings test on labor supply and pension benefit claims

Tomoki KITAMURA and Yoshimi ADACHI

Online Appendix

Table A1. Description of supplemental files.

	File name	Description
1	data_2pone.xlsx	Dataset file for main analysis (Excel format)
2	data_2pone.dta	Dataset file for main analysis (STATA format). This file requires STATA to open.
3	10_reg_20240320_1pone_1.do	STATA code (STATA do file) for main analysis, which can replicate Tables 3–7. This file requires STATA to open.
4	10_reg_20240320_1pone_1.txt	STATA code for main analysis (text file)
5	data_3cs4pone.xlsx	Data file for mixed logit analysis (Excel format)
6	data_3cs4pone.dta	Data file for mixed logit analysis (STATA format). This file requires STATA to open.
7	25_mixlogit20240224_5pone.do	STATA code (STATA do file) for mixed logit analysis, which can replicate Table 9. This file requires STATA to open.
8	25_mixlogit20240224_5pone.txt	STATA code for mixed logit analysis (text file)

Table A1 lists the files attached to this supplemental information. The datasets for the main analysis are contained in “data_2pone.xlsx” and “data_2pone.dta”, which include all data used to generate Tables 3–7. The file “data_2pone.dta” is compatible with STATA, while “data_2pone.xlsx” is available in Excel format for users of different software. The STATA do-file “10_reg_20240320_1pone_1.do” contains commands for the statistical analysis to replicate Tables 3–7, with “10_reg_20240320_1pone_1.txt” providing an equivalent text file version.

The datasets for the mixed logit analysis are provided in “data_3cs4pone.xlsx” (Excel format) and “data_3cs4pone.dta” (STATA format). The file “25_mixlogit20240224_5pone.do” in STATA can replicate Table 9, and “25_mixlogit20240224_5pone.txt” serves as its text file counterpart.

Files requiring STATA, such as “data_2pone.dta”, “10_reg_20240320_1pone_1.do”, “data_3cs4pone.dta”, and “25_mixlogit20240224_5pone.do”, are noted. Users without STATA should refer to the Excel and text files for compatibility.

Table A2. Experimental instruction for the FP treatment.

The pension plan has been revised.

The numbers in blue are different from the previous question. Please look at them carefully and answer the question. Other figures are the same as the last question.

Suppose that you are now 59 years old. You are considering whether to retire at age 65 or to work at 65 and retire at 66. Which work styles and ways of receiving the public pension (the sum of the EPI and BP) would you prefer? Please choose the option that best applies to you.

		Plan A	Plan B	Plan C	Plan D	Plan E
Age 65	Labor income	665	665	443	443	0
	Pension benefits	88	0	198	0	198
Age 66 or older	Labor income	0	0	0	0	0
	Pension benefits	198	**215**	198	215	198
Your choice		○	○	○	○	○

Unit: Annual JPY 10,000

Explanation of work plan

- In Plan A (HIWP: high-income with pension benefits), you obtain an annual labor income of JPY 6.65 million (M) at 65 with annual public pension benefits of JPY 0.88 M. You retire at 66 and obtain public pension benefits of JPY 1.98 M annually.
- In Plan B (HINP: high-income without pension benefits), you obtain an annual labor income of JPY 6.65 M at 65 without public pension benefits. You retire at 66 and obtain public pension benefits of JPY 2.15 M annually.
- In Plan C (LIWP: low-income with pension benefits), you obtain an annual labor income of JPY 4.43 M at 65 by reducing work hours with annual public pension benefits of JPY 1.98 M. You retire at 66 and obtain annual public pension benefits of JPY 1.98 M.
- In Plan D (LINP: low-income without pension benefits), you obtain an annual labor income of JPY 4.43 M at 65 by reducing work hours without public pension benefits. You retire at 66 and obtain annual public pension benefits of JPY 2.15 M.
- In Plan E (No work), you retire at 65 and obtain annual public pension benefits of JPY 1.98 M.

Note: JPY 1M is approximately USD 8330.00. Symbols in parentheses, such as HIWP, are intended for readers of this paper. Such explanations were excluded from the survey. The actual explanation is provided in Japanese.

Table A3. Experimental instruction for the CP treatment.

The pension plan has been revised.

The numbers in blue are different from the previous question. Please look at them carefully and answer the question. Other figures are the same as the last question.

Suppose that you are now 59 years old. You are considering whether to retire at age 65 or to work at 65 and retire at 66. Which work styles and ways of receiving the public pension (the sum of the EPI and BP) would you prefer? Please choose the option that best applies to you.

		Plan A	Plan B	Plan C	Plan D	Plan E
Age 65	Labor income	665	665	443	443	0
	Pension benefits	**198**	0	198	0	198
Age 66 or older	Labor income	0	0	0	0	0
	Pension benefits	198	205	198	215	198
Your choice		○	○	○	○	○

Unit: Annual JPY 10,000

Explanation of work plan

- In Plan A (HIWP: high-income with pension benefits), you obtain an annual labor income of JPY 6.65 M at 65 with annual public pension benefits of JPY 1.98 M. You retire at 66 and obtain public pension benefits of JPY 1.98 M annually.
- In Plan B (HINP: high-income without pension benefits), you obtain an annual labor income of JPY 6.65 M at 65 without public pension benefits. You retire at 66 and obtain public pension benefits of JPY 2.05 M annually.
- In Plan C (LIWP: low-income with pension benefits), you obtain an annual labor income of JPY 4.43 M at 65 by reducing work hours with annual public pension benefits of JPY 1.98 M. You retire at 66 and obtain annual public pension benefits of JPY 1.98 M.
- In Plan D (LINP: low-income without pension benefits), you obtain an annual labor income of JPY 4.43 M at 65 by reducing work hours without public pension benefits. You retire at 66 and obtain annual public pension benefits of JPY 2.15 M.
- In Plan E (No work), you retire at 65 and obtain annual public pension benefits of JPY 1.98 M.

Note: JPY 1M is approximately USD 8330.00. Symbols in parentheses, such as HIWP, are intended for readers of this paper. Such explanations were excluded from the survey. The actual explanation is provided in Japanese.

Table A4. Experimental instruction for the FPCP treatment.

The pension plan has been revised.

The numbers in blue are different from the previous question. Please look at them carefully and answer the question. Other figures are the same as the last question.

Suppose that you are now 59 years old. You are considering whether to retire at age 65 or to work at 65 and retire at 66. Which work styles and ways of receiving the public pension (the sum of the EPI and BP) would you prefer? Please choose the option that best applies to you.

		Plan A	Plan B	Plan C	Plan D	Plan E
Age 65	Labor income	665	665	443	443	0
	Pension benefits	**198**	0	198	0	198
Age 66 or older	Labor income	0	0	0	0	0
	Pension benefits	198	**215**	198	215	198
Your choice		○	○	○	○	○

Unit: Annual JPY 10,000

Explanation of work plan

- In Plan A (HIWP: high-income with pension benefits), you obtain an annual labor income of JPY 6.65 M at 65 with annual public pension benefits of JPY 1.98 M. You retire at 66 and obtain public pension benefits of JPY 1.98 M annually.
- In Plan B (HINP: high-income without pension benefits), you obtain an annual labor income of JPY 6.65 M at 65 without public pension benefits. You retire at 66 and obtain public pension benefits of JPY 2.15 M annually.
- In Plan C (LIWP: low-income with pension benefits), you obtain an annual labor income of JPY 4.43 M at 65 by reducing work hours with annual public pension benefits of JPY 1.98 M. You retire at 66 and obtain annual public pension benefits of JPY 1.98 M.
- In Plan D (LINP: low-income without pension benefits), you obtain an annual labor income of JPY 4.43 M at 65 by reducing work hours without public pension benefits. You retire at 66 and obtain annual public pension benefits of JPY 2.15 M.
- In Plan E (No work), you retire at 65 and obtain annual public pension benefits of JPY 1.98 M.

Note: JPY 1M is approximately USD 8330.00. Symbols in parentheses, such as HIWP, are intended for readers of this paper. Such explanations were excluded from the survey. The actual explanation is provided in Japanese.
