# Supplementary material for: Impact of eliminating retirement earnings test on labor supply and pension benefit claims
Source: PLoS One. 2024 Aug 9;19(8):e0304458. doi: 10.1371/journal.pone.0304458 (PMC11315313; doi:10.1371/journal.pone.0304458)
Supplement: S1 Appendix — (DOCX) [file pone.0304458.s002.docx]

**Appendices**

**Table A1. Detailed responses for categorical individual characteristics.**

| **Panel A. Responses regarding trust in public pension.** | | | |
| --- | --- | --- | --- |
|  | Number of observations | Proportion | Cumulative  proportion |
| Almost unreliable | 137 | 12.91 | 12.91 |
| Very unreliable | 246 | 23.19 | 36.1 |
| Rather unreliable | 276 | 26.01 | 62.11 |
| Rather reliable | 271 | 25.54 | 87.65 |
| Fairly reliable | 106 | 9.99 | 97.64 |
| Quite reliable | 25 | 2.36 | 100 |
|  |  |  |  |
| **Panel B. Responses regarding survival probability at 85.** | | | |
|  | Number of observations | Proportion | Cumulative  proportion |
| Less than 5% | 215 | 20.26 | 20.26 |
| 5% | 38 | 3.58 | 23.85 |
| 10% | 108 | 10.18 | 34.02 |
| 15% | 36 | 3.39 | 37.42 |
| 20% | 82 | 7.73 | 45.15 |
| 25% | 26 | 2.45 | 47.6 |
| 30% | 109 | 10.27 | 57.87 |
| 35% | 22 | 2.07 | 59.94 |
| 40% | 40 | 3.77 | 63.71 |
| 45% | 10 | 0.94 | 64.66 |
| 50% | 190 | 17.91 | 82.56 |
| 55% | 6 | 0.57 | 83.13 |
| 60% | 32 | 3.02 | 86.15 |
| 65% | 8 | 0.75 | 86.9 |
| 70% | 36 | 3.39 | 90.29 |
| 75% | 9 | 0.85 | 91.14 |
| 80% | 38 | 3.58 | 94.72 |
| 85% | 6 | 0.57 | 95.29 |
| 90% | 16 | 1.51 | 96.8 |
| 95% | 1 | 0.09 | 96.89 |
| More than 95% | 33 | 3.11 | 100 |
|  |  |  |  |
| **Panel C. Responses regarding health condition.** | | | |
|  | Number of observations | Proportion | Cumulative  proportion |
| Poor | 10 | 0.94 | 0.94 |
| Fairly Poor | 67 | 6.31 | 7.26 |
| Somewhat poor | 145 | 13.67 | 20.92 |
| Somewhat good | 377 | 35.53 | 56.46 |
| Fairly Good | 349 | 32.89 | 89.35 |
| Good | 113 | 10.65 | 100 |

**Table A2. The difference in individual characteristics between our respondents and population.**

| **Panel A. Household head’s income.** | | | | | | |
| --- | --- | --- | --- | --- | --- | --- |
|  | 40-44 | 45-49 | 50-54 | 55-59 | 60-64 | 65-69 |
| Population | 5.9 | 6.3 | 7.0 | 6.8 | 4.2 | 2.5 |
| Our sample | 7.0 | 7.1 | 6.9 | 6.9 |  |  |
|  |  |  |  |  |  |  |
| **Panel B. Financial asset holdings.** | | | | | | |
|  | 40-44 | 45-49 | 50-54 | 55-59 | 60-64 | 65-69 |
| Population | 8.6 | 10.3 | 13.2 | 16.1 | 18.7 | 16.1 |
| Our sample | 7.2 | 8.3 | 9.3 | 10.5 |  |  |
|  |  |  |  |  |  |  |
| **Panel C. House own.** | | | | | | |
|  | 40-44 | 45-49 | 50-54 | 55-59 | 60-64 | 65-69 |
| Population | 55% | 60% | 65% | 71% | 76% | 79% |
| Our sample | 68% | 77% | 72% | 74% |  |  |
|  |  |  |  |  |  |  |
| **Panel D. University graduates.** | | | | | | |
|  | 40-44 | 45-49 | 50-54 | 55-59 |  |  |
| Population | 42% | 40% | 42% | 45% |  |  |
| Our sample | 80% | 80% | 67% | 73% |  |  |

**Note:** **The units for both Panel A and Panel B are in JPY million, with population data sampled from the National Survey of Family Income and Expenditure conducted in 2019 (https://www.stat.go.jp/data/zenkokukakei/2019/index.html). Panel C’s population data are drawn from the Housing and Land Survey of 2018 (https://www.stat.go.jp/data/jyutaku/). Lastly, the population for Panel D is based on the Employment Status Survey from 2017 (https://www.stat.go.jp/data/shugyou/2017/index.html).**

**Table A3. Individual characteristics according to respondent groups.**

|  |  | Group 1 | | Group 2 | | Group 3 | | Difference | | | Difference | | | Joint | |
| --- | --- | --- | --- | --- | --- | --- | --- | --- | --- | --- | --- | --- | --- | --- | --- |
|  |  |  |  |  |  |  |  | Group 2-1 | | | Group 2-1 | | | Difference | |
|  |  | Mean | SE | Mean | SE | Mean | SE | EST | SE |  | EST | SE |  | F-test | |
| (1) | Trust for public pension | 3.06 | (0.07) | 2.98 | (0.06) | 3.07 | (0.07) | -0.07 | (0.10) |  | 0.01 | (0.10) |  | 0.60 |  |
| (2) | Survival probability at age 85 | 31.88 | (1.41) | 33.55 | (1.45) | 32.11 | (1.43) | 1.66 | (2.02) |  | 0.22 | (2.01) |  | 0.68 |  |
| (3) | Financial literacy | 0.58 | (0.02) | 0.58 | (0.02) | 0.61 | (0.02) | -0.00 | (0.03) |  | 0.03 | (0.03) |  | 0.01 |  |
| (4) | Health condition | 4.18 | (0.06) | 4.33 | (0.06) | 4.25 | (0.06) | 0.15 | (0.08) | * | 0.07 | (0.08) |  | 3.52 | * |
| (5) | University | 0.76 | (0.02) | 0.73 | (0.02) | 0.76 | (0.02) | -0.03 | (0.03) |  | 0.00 | (0.03) |  | 0.81 |  |
| (6) | Small company | 0.50 | (0.03) | 0.47 | (0.03) | 0.52 | (0.03) | -0.04 | (0.04) |  | 0.02 | (0.04) |  | 0.95 |  |
| (7) | Mandatory retirement age 65 or more | 0.41 | (0.03) | 0.32 | (0.02) | 0.31 | (0.02) | -0.09 | (0.04) | ** | -0.09 | (0.04) | *** | 6.21 | ** |
| (8) | Household income | 7.10 | (0.10) | 6.94 | (0.09) | 6.89 | (0.09) | -0.16 | (0.13) |  | -0.21 | (0.13) |  | 1.44 |  |
| (9) | Respondent's income | 5.89 | (0.09) | 5.75 | (0.08) | 5.77 | (0.09) | -0.14 | (0.12) |  | -0.12 | (0.12) |  | 1.27 |  |
| (10) | Financial assets | 9.80 | (1.02) | 8.34 | (0.99) | 8.60 | (0.78) | -1.46 | (1.42) |  | -1.20 | (1.28) |  | 1.05 |  |
| (11) | House own without mortgage | 0.28 | (0.02) | 0.23 | (0.02) | 0.24 | (0.02) | -0.05 | (0.03) |  | -0.04 | (0.03) |  | 2.31 |  |
| (12) | House own with mortgage | 0.43 | (0.03) | 0.52 | (0.03) | 0.52 | (0.03) | 0.09 | (0.04) | ** | 0.09 | (0.04) | ** | 5.87 | ** |
| (13) | House rent | 0.30 | (0.02) | 0.26 | (0.02) | 0.25 | (0.02) | -0.04 | (0.03) |  | -0.05 | (0.03) |  | 1.49 |  |
| (14) | Age | 50.16 | (0.28) | 49.99 | (0.30) | 49.80 | (0.30) | -0.16 | (0.41) |  | -0.36 | (0.42) |  | 0.16 |  |
|  | N | 352 |  | 354 |  | 355 |  |  |  |  |  |  |  |  |  |

**Note**: Standard errors are estimated based on the delta method. ***, **, and * indicate statistical significance at p < 0.01, p < 0.05, and p < 0.1, respectively.

**Table A4. Individual characteristics according to respondent groups.**

| **Panel A. Effect of eliminating earnings test according to respondent group.** | | | | | | | | | | |
| --- | --- | --- | --- | --- | --- | --- | --- | --- | --- | --- |
|  |  | Question | (1) | | (2) | | (3) | | (4) | |
| Group | Treatment | order | HIWP | | HINP | | LIWP | | LINP | |
| Group 1 | Control | 1 | 0.206 |  | 0.283 |  | 0.220 |  | 0.213 |  |
|  |  |  | (0.021) |  | (0.024) |  | (0.022) |  | (0.022) |  |
|  | FP | 2 | 0.112 |  | 0.556 |  | 0.158 |  | 0.108 |  |
|  |  |  | (0.017) |  | (0.026) |  | (0.019) |  | (0.017) |  |
|  | CP | 3 | 0.427 |  | 0.198 |  | 0.152 |  | 0.165 |  |
|  |  |  | (0.026) |  | (0.022) |  | (0.019) |  | (0.020) |  |
|  | FPCP | 4 | 0.299 |  | 0.394 |  | 0.144 |  | 0.105 |  |
|  |  |  | (0.024) |  | (0.026) |  | (0.019) |  | (0.016) |  |
| Group 2 | Control | 1 | 0.198 |  | 0.262 |  | 0.230 |  | 0.221 |  |
|  |  |  | (0.021) |  | (0.023) |  | (0.022) |  | (0.022) |  |
|  | FP | 3 | 0.181 |  | 0.462 |  | 0.205 |  | 0.077 |  |
|  |  |  | (0.020) |  | (0.026) |  | (0.022) |  | (0.014) |  |
|  | CP | 2 | 0.450 |  | 0.140 |  | 0.168 |  | 0.176 |  |
|  |  |  | (0.026) |  | (0.019) |  | (0.020) |  | (0.020) |  |
|  | FPCP | 4 | 0.337 |  | 0.372 |  | 0.154 |  | 0.068 |  |
|  |  |  | (0.025) |  | (0.026) |  | (0.019) |  | (0.014) |  |
| Group 3 | Control | 1 | 0.179 |  | 0.289 |  | 0.233 |  | 0.231 |  |
|  |  |  | (0.020) |  | (0.024) |  | (0.022) |  | (0.022) |  |
|  | FP | 3 | 0.156 |  | 0.506 |  | 0.174 |  | 0.084 |  |
|  |  |  | (0.019) |  | (0.026) |  | (0.020) |  | (0.015) |  |
|  | CP | 4 | 0.415 |  | 0.213 |  | 0.152 |  | 0.146 |  |
|  |  |  | (0.026) |  | (0.021) |  | (0.019) |  | (0.019) |  |
|  | FPCP | 2 | 0.261 |  | 0.418 |  | 0.157 |  | 0.095 |  |
|  |  |  | (0.023) |  | (0.026) |  | (0.019) |  | (0.016) |  |
|  |  |  |  |  |  |  |  |  |  |  |
| **Panel B. Difference in group.** | | | | | | | | | | |
|  |  |  | (1) | | (2) | | (3) | | (4) | |
| Difference | Treatment |  | HIWP | | HINP | | LIWP | | LINP | |
| Group 2-1 | Control |  | -0.007 |  | -0.022 |  | 0.010 |  | 0.008 |  |
|  |  |  | (0.030) |  | (0.034) |  | (0.032) |  | (0.031) |  |
|  | FP |  | 0.070 | *** | -0.094 | ** | 0.047 |  | -0.031 |  |
|  |  |  | (0.026) |  | (0.037) |  | (0.029) |  | (0.022) |  |
|  | CP |  | 0.023 |  | -0.058 | ** | 0.016 |  | 0.011 |  |
|  |  |  | (0.037) |  | (0.029) |  | (0.028) |  | (0.028) |  |
|  | FPCP |  | 0.037 |  | -0.022 |  | 0.010 |  | -0.037 | * |
|  |  |  | (0.035) |  | (0.037) |  | (0.027) |  | (0.021) |  |
| Group 3-1 | Control |  | -0.027 |  | 0.005 |  | 0.013 |  | 0.018 |  |
|  |  |  | (0.030) |  | (0.034) |  | (0.032) |  | (0.031) |  |
|  | FP |  | 0.045 | * | -0.050 |  | 0.016 |  | -0.024 |  |
|  |  |  | (0.026) |  | (0.037) |  | (0.028) |  | (0.022) |  |
|  | CP |  | -0.012 |  | 0.015 |  | -0.001 |  | -0.019 |  |
|  |  |  | (0.037) |  | (0.031) |  | (0.027) |  | (0.027) |  |
|  | FPCP |  | -0.039 |  | 0.024 |  | 0.013 |  | -0.010 |  |
|  |  |  | (0.034) |  | (0.037) |  | (0.027) |  | (0.023) |  |

**Note**: Standard errors are estimated based on the delta method. ***, **, and * indicate statistical significance at p < 0.01, p < 0.05, and p < 0.1, respectively.
